# Vertebral Subluxation and Systems Biology: An Integrative Review Exploring the Salutogenic Influence of Chiropractic Care on the Neuroendocrine-Immune System

**DOI:** 10.7759/cureus.56223

**Published:** 2024-03-15

**Authors:** Amy Haas, Jonathan Chung, Christopher Kent, Brooke Mills, Matthew McCoy

**Affiliations:** 1 Research, Foundation for Vertebral Subluxation, Kennesaw, USA; 2 Research, Sherman College, Spartanburg, USA

**Keywords:** evidence informed practice, chiropractic adjustment, adaptability, salutogenesis, integrative physiology, chiropractic care, vertebral subluxation

## Abstract

In this paper we synthesize an expansive body of literature examining the multifaceted influence of chiropractic care on processes within and modulators of the neuroendocrine-immune (NEI) system, for the purpose of generating an inductive hypothesis regarding the potential impacts of chiropractic care on integrated physiology. Taking a broad, interdisciplinary, and integrative view of two decades of research-documented outcomes of chiropractic care, inclusive of reports ranging from systematic and meta-analysis and randomized and observational trials to case and cohort studies, this review encapsulates a rigorous analysis of research and suggests the appropriateness of a more integrative perspective on the impact of chiropractic care on systemic physiology. A novel perspective on the salutogenic, health-promoting effects of chiropractic adjustment is presented, focused on the improvement of physical indicators of well-being and adaptability such as blood pressure, heart rate variability, and sleep, potential benefits that may be facilitated through multiple neurologically mediated pathways. Our findings support the biological plausibility of complex benefits from chiropractic intervention that is not limited to simple neuromusculoskeletal outcomes and open new avenues for future research, specifically the exploration and mapping of the precise neural pathways and networks influenced by chiropractic adjustment.

## Introduction and background

A primary function of the human body is to adapt to its environment in order to survive and propagate the species. The process of adaptation requires a patent neurological capacity to (1) rapidly and accurately sense threats to or changes in the body’s internal and external environments; (2) integrate multiple forms of sensory0 information; and (3) execute a coherent, physiologically coordinated and temporally appropriate multi-system response. The human nervous system must, therefore, be capable of sensitively perceiving and accurately integrating all forms of information from its environment including physical changes, chemical changes, or emotional/social challenges, as it continually assesses and overcomes potential threats [1]. Further, it must also be capable of generating a coordinated response via a symphony of body systems, promoting coherent maintenance of adaptation within the body’s dynamic equilibrium and safeguarding the body’s ability to thrive under diverse circumstances [2,3].

Integrative systems biology, or integrative physiology [4], is the study of coordinated responses from functionally coupled systems such as the nervous system, the endocrine system, and the immune system, which reciprocally influence one another [5-8]. The growth in the body of research outlining the connections between these subsystems has been exponential in the past century, and has overcome the now-historical notion that the central nervous system (CNS) is “immunologically pristine” and separated from the immune system [9,10]. While the relationship between the nervous and endocrine systems has long been accepted [11], current research has elucidated and defined numerous physical and functional conduits by which the nervous system and immune system interact and achieve coherent synergy. These conduits include direct interface of the immune system with both the sympathetic and parasympathetic divisions of the autonomic nervous system [5,12-14], direct innervation and control of immune structures, including neural circuits that directly modulate antigen flow through lymph nodes [5,15,16], bidirectional interactions at the dural neuro-immune interface within the CNS [10], and shared modulation by neurotransmitters, neuropeptides, hormones, and other small molecule messengers [17-19]. Current hypotheses in the field of neuroendocrine immunology hold that neuroimmune circuits may also mediate and modulate inter-organ communication, thereby serving as the primary conductor in the coordination of human physiology as a whole [20]. In the neuroendocrine-immune (NEI) system, each of the three component arms - nervous system, immune system, and endocrine system - modulates one another via multiple means of “crosstalk,” in order to achieve synergistic and integrated physiological changes and coordinated adaptation to an environmental stressor.

This growing body of research on the reciprocal nature of communication between the subsystems of the NEI supersystem underscores the importance of integrative physiology research [21]. Understanding the coordinated synergy between these systems is critical for an appreciation of how the human body functions as a whole: one must maintain an appreciation that an input that affects one subsystem would be naturally expected to impact or elicit a coordinated adaptive response from related subsystems. In the nascent exploration of integrative biology, considering and examining global, multi-system responses resulting from any impetus to each subsystem is not only appropriate, but it is also requisite.

In its varied forms, physical healthcare utilizes numerous approaches intended to improve and support human adaptation and life. These approaches include interventions that range from preventive care to emergency intervention, and from drug/surgical treatment to “holistic” interventions that include nutrition and exercise [22]. Common to many healthcare approaches is the concept that with one specific and directed intervention, changes in multiple physiologic systems beyond the one originally targeted can be observed, given the interconnected nature of the body’s component systems [23]. As the field of dynamical systems biology rapidly develops, practitioners across many healthcare disciplines are paying heed to interrelated interventional effects, both direct and indirect, with an eye towards truly integrative and collaborative healthcare.

Chiropractic care is a profession dedicated to the conservative, non-surgical care of the spine by way of spinal adjustment, with the goal of improving spinal structure and function in order to support the body’s natural ability to heal itself [24]. Within the field of chiropractic spinal care, a chasm exists between practitioners who consider benefits limited to neuromusculoskeletal (NMS) effects, and practitioners who hold that effects of the chiropractic adjustment supersede NMS outcomes [25]. The latter subset of chiropractors address the spine specifically to locate and correct the vertebral subluxation (VS), which is thought to introduce interference to afferent and/or efferent flow within the nervous system, thereby evoking peripheral physiological maladaptations [26,27]. Correction of VS is held to promote salutogenesis, defined as the creation or restoration, promotion, and maintenance of well-being, adaptability, and resilience in the face of challenge [28]. Salutogenesis is manifested physically by the enhancement of coordinated adaptation of integrated body systems thereby mitigating the effects of allostatic load [28,29]. Herein, we explore integrative physiology outcomes indicative of enhancement of physical well-being in chiropractic research in order to elucidate a body of evidence demonstrating that this ostensibly NMS-directed intervention elicits adaptive salutogenic change from multiple systems. It should be noted that an exploration of the impact of a physical interventional approach on interconnected physiologic systems is not exclusive to the field of chiropractic care. This concept has been approached in related fields, including osteopathy and acupuncture [30-36]. While the precise mode of physical intervention and philosophical intent clearly differs amongst these disciplines, the fields of chiropractic care, osteopathy, and acupuncture share a commonality: each represents an NMS intervention that evokes somatosensory and/or autonomic input to the CNS, that may result in changes to both NMS outcomes and additional integrative physiology outcomes that are not categorized as NMS. Therefore these allied fields may be considered together as a genre, under a hypothesis that neural input from diverse physical or manual stimuli may lead to multiple outcomes in an integrative physiology-centric model. From the perspective of the chiropractic profession, the nature of the NMS input generated or modulated by the chiropractic adjustment is of both theoretical and practical importance: whether the adjustment has strictly local NMS effects, or if VS and adjustment thereof have afferent and/or efferent impacts with resultant adaptive change in integrated physiology outcomes, is at the center of a longstanding debate. A consideration of the integrative physiology outcomes of chiropractic care for VS that supersede NMS outcomes observed with spinal manipulation is consistent with established findings from related fields, and opens greater possibilities for research on multi-system and salutogenic outcomes of chiropractic care.

Evidence-based healthcare is defined as a process of integrating clinical expertise in the form of critical reasoning (acquired through training and professional experience) with unique client perspectives and needs, and weighing these factors with the best available evidence in published scientific literature to execute clinical decision-making [37]. Such evidence is traditionally categorized within a hierarchical continuum, ranging from meta-analyses (highest) to expert opinion (lowest) [38]. The more traditional approach of evidence-based care prioritizes and/or limits patient care recommendations to be based on conclusions gleaned only from the upper echelons of this hierarchy (randomized controlled trials (RCTs) and higher); however, sometimes RCTs are not feasible, and practical application of RCTs to personalized recommendations may or may not be possible, as distinctions may exist between idealized trial cohorts and actual clinical presentations that limit comparability [39-41]. As Daramola and Rhee state in their essay for the *AMA Journal of Ethics*, “One must be careful not to adopt an inflexible approach of only applying recommendations of greater strength. The practice of evidence-based medicine is not 'cookbook' medicine, and therefore the basis for patient care decisions should not be restricted to randomized trials or meta-analyses” [42,43]. Evidence-informed practice (EIP) was conceptualized as an update to the historical evidence-based practice (EBP) model, in order to consider evidence along the entire continuum, and is inclusive of observational, cohort, and case reports as evidence to be considered in the clinical decision-making process [40,44,45]. The decision of whether to adopt an evidence-based or an evidence-informed scope of practice is a point of paramount significance for how chiropractors are allowed by regulatory agencies to practice, in North America and worldwide. To this end, the present broad-sweep integrative review was conducted to compile and discuss multiple levels of evidence for a variety of salutogenic integrative physiology outcomes for chiropractic care, including not only RCTs but also quantitative observational, cohort, and case-study evidence, using keywords for well-characterized NEI modulators, in order to empower the practice of evidence-informed chiropractic care.

Towards greater recognition of all levels of evidence

We present here a compiled database of diverse physiological outcomes reported with chiropractic care, inclusive of diverse methodologies of quantitative and objective evidence, that supports the application of chiropractic adjustment for the promotion of optimal health and function (salutogenesis). The results of these searches are considered in light of the growing field of integrative physiology, and we present several biologically plausible mechanisms that may explain the salutogenic outcomes observed for chiropractic care. The contents of this work may inform the chiropractic field practitioner of multiple levels and types of evidence to be considered in their evidence-informed clinical decision-making process, and may also inform future research agendas for the chiropractic profession.

## Review

Literature search: Methods

The survey of literature was limited to a search of two databases, PubMed (Medline) and Index to Chiropractic Literature (ICL), for the sake of feasibility. Keywords “Chiropractic” or “Spinal Manipulation,” both of which are commonly used in the research literature to describe chiropractic care as a genre, were cross-referenced with the following keywords or terms (Table 1):

**Table 1:** Keyword search terms

Autoimmune	Blood pressure	Cortisol	Cytokine
Heart Rate Variability	Immune/Immunity	Interferon	Interleukin
Sleep	Substance P	Transforming Growth Factor	Tumor Necrosis Factor

Inclusion and exclusion criteria

Inclusion criteria include both keywords searched, peer-reviewed, published level VIII and stronger evidence, narrative reviews, quantitative evidence, and publications from 2000 to 2022. Excluded criteria consist of expert opinion (level IX evidence), poster presentations/abstracts, animal studies, null studies or qualitative evidence, and publications from 1999 and prior.

Entries were accepted for further assessment if they contained either “spinal manipulation” or “chiropractic” in conjunction with one of the 12 keywords in Table 1. Using Stony Brook University Library’s Levels of Evidence pyramid level VIII or higher evidence was accepted for further assessment. In this hierarchy, Level VIII comprises evidence from non-RCTs, non-randomized clinical trials, cohort studies, case series, case reports, and individual qualitative studies. The next higher level, Level VI, comprises randomized clinical trials, and levels V and higher are meta- and systematic analyses of RCTs. Level IX, expert opinion, was excluded in our search due to the lack of original and/or quantitative data intrinsic to these reports.

**Figure 1:**
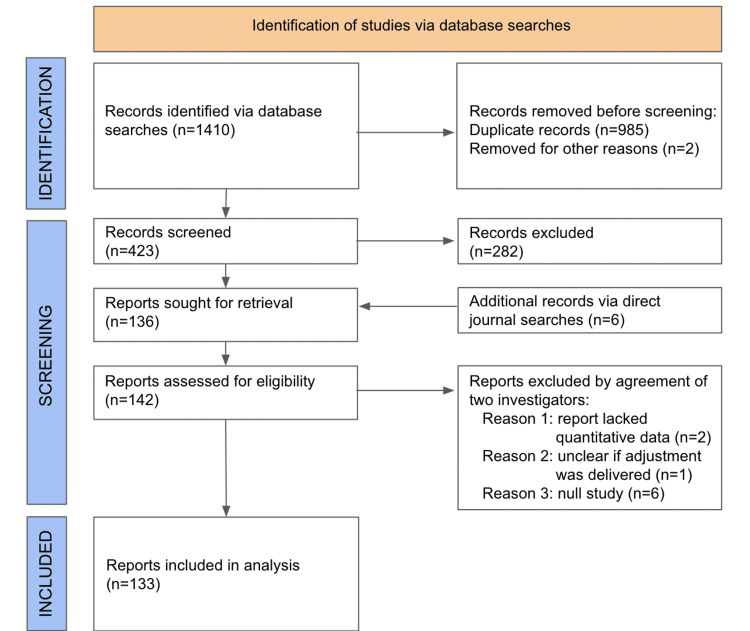
Flowchart representing literature search A total of 1410 references were identified via database searches of PubMed (MEDLINE) and ICL (Index to Chiropractic Literature). After the removal of duplicates and screening of titles and abstracts using inclusion/exclusion criteria, and the addition of six records identified from direct journal searching, a total of 142 records were sought for retrieval. Nine papers were excluded by mutual agreement (A. Haas and J. Chung) as follows: two papers did not offer quantitative outcomes measures; one paper did not clearly include chiropractic adjustment or manipulation in their methods; six papers did not report statistically significant changes and therefore did not meet inclusion criteria. The resultant final database contains 133 references mutually agreed upon by two investigators (A. Haas and J. Chung) as meeting the inclusion/exclusion criteria for the present study.

Figure 1 presents the flowchart of the selection process.

Each keyword search was performed using Advance Search Modality (PubMed) or Advanced Search Builder (ICL). Date parameters were set to 2000 through the end of 2022; literature published prior to 2000 was excluded, since these data would be expected to be included in reviews and meta-analyses falling within the date range selected. All searches were completed in January 2023. The identified references were exported in separate batches, per keyword and either Chiropractic or Spinal Manipulation to compile the preliminary dataset in Table 2.

**Table 2:** Preliminary dataset, records identified via PubMed and ICL (Index to Chiropractic Literature) keyword searches (includes duplicates)

Keyword	Chiropractic		Spinal manipulation		Total references per batch
	PubMed	ICL	PubMed	ICL	
Autoimmune	35	14	26	1	76
Blood Pressure	71	74	78	26	249
Cortisol	7	10	10	4	31
Cytokine	29	5	69	29	110
HRV or Heart Rate Variability	27	53	30	23	133
Immune or Immunity	93	94	84	55	326
Interferon	6	2	8	2	18
Interleukin	18	9	29	10	66
Sleep	87	152	33	27	299
Substance P	5	0	13	0	18
TGF or Transforming Growth Factor	4	1	8	1	15
TNF or Tumor Necrosis Factor	14	16	25	15	70
TOTAL					1410

Keyword batches were then merged (ex., “chiropractic” and “autoimmune” PubMed search results were merged with “chiropractic” and “autoimmune” ICL search results) and 985 duplicate entries were removed. A preliminary screen for inclusion/exclusion criteria was then performed on the remaining 423 records based on article titles and abstracts for each keyword batch and 282 records were excluded.

The resultant batches per keyword for “Chiropractic” and “Spinal Manipulation” were then merged to create a final list of records per keyword. Six articles were added to this database after manual search of articles published in 2022 but not yet indexed in ICL from the following journals: *Annals of Vertebral Subluxation Research*, *Journal of Pediatric, Maternal, & Family Health*, and *Journal of Upper Cervical Chiropractic Research*. A total of 142 reports were accessed and critically assessed in detail for inclusion/exclusion criteria per content by two investigators (A. Haas and J. Chung). Disagreements on inclusion were settled by mutual investigator agreement, and nine additional reports were excluded at this step from the dataset as not fully meeting inclusion/exclusion criteria, leaving 133 reports in the final dataset. Table 3 below contains this compendium of research articles organized by keyword category, inclusive of both chiropractic and spinal manipulation. In sum, the search and refinement process yielded 133 unique reports [46-178] either containing or reviewing quantitative data that demonstrate the impact of the chiropractic adjustment/spinal manipulation on various NEI markers and processes and are therefore represented multiple times within Table 3.

**Table 3:** Final dataset of references, 2000-2022, demonstrating quantitative evidence of the impact of chiropractic care on neuroimmune-endocrine modulators or outcomes, per keyword.

Search keyword	Total unique articles that meet criteria	REFS**
Autoimmune	0	n/a
Blood Pressure	30	[46-75]
Cortisol	11	[76-86]
Cytokine	12	[84,85,87-96]
Heart Rate Variability/HRV	29	[63,64,70,73,97-121]
Immune/Immunity	27	[85,93,96,122-145]
Interferon	2	[87,95]
Interleukin	10	[84-87,90-92,95,137,138]
Sleep	29	[146-174]
Substance P	4	[86,175-177]
Tumor Necrosis Factor/TNF	6	[88,89,92,95,96,178]
Transforming Growth Factor/TGF	0	n/a

Review of included references from the literature search

The present integrative literature review was undertaken to explore an integrative physiology perspective on how and why spinal adjustments elicit a neuroimmune-endocrine response within the human body, with the potential for broad-reaching clinical effects and outcomes. As such, this review was not designed to provide a comprehensive meta-analysis or systematic review of isolated areas within the chiropractic field, but rather to consider the interconnected nature of the findings within this body of literature. Each of the 133 references identified via the literature search offers observational and/or clinical data demonstrating that spinal adjustments affect the NEI supersystem, its component subsystems, or biomarkers/effectors common to these subsystems in measurable ways. Considered together, this body of literature provides supportive evidence that spinal adjustments modulate NEI supersystem-mediated clinical outcomes.

For the present literature search, the category terms employed were “spinal manipulation” and “chiropractic adjustment,” with the rationale that chiropractic care (which may be labeled in research reports “spinal manipulation,” “SMT,” “chiropractic adjustment,” “spinal adjustment,” or “chiropractic manipulation”) is a broad category of manual, non-surgical, spinal intervention, all of which may have effects on aspects of the NEI supersystem. The present work focuses on the exploration of the biological plausibility of how neural input resulting from chiropractic intervention to the spine affects the NEI supersystem. However, considering chiropractic care as a broad genre also created the opportunity to explore characteristics that create distinctions between each of these subtypes of chiropractic care as delineated above. Philosophically, “spinal manipulation” and “chiropractic adjustment” within this broad category of chiropractic care bear a critical methodological distinction that will be highlighted in the following sections, as it portends important implications for the design and interpretation of research trials. Spinal manipulation methods, employed by numerous professions including physiotherapists, osteopaths, and some chiropractors, are often targeted at arbitrary regions of the spine (“manipulable lesions”), and are intended to reduce pain and improve general spinal or regional mobility. In contrast, the specific chiropractic adjustment, employed by a different subset of the chiropractic profession, is a targeted intervention directed at a discrete spinal segment (or segments) that exhibits characteristics of VS. VS is a specific clinical spinal abnormality that perturbs the normal intervertebral relationships of one or more articulations of the spinal column or the immediate weight-bearing components of the axial skeleton, is accompanied by a change in the morphology of the tissue occupying the neural canal and/or intervertebral foramina, and alters neural function sufficiently to interfere with the transmission of organizing information [179,180]. The neurological derangements characteristic of VS may include dysafferentation, dysponesis, dysautonomia, changes to neuroplasticity, and changes to ephaptic transmission [181]. VS can be reproducibly and reliably measured by observation and quantification of abnormal spinal position and biomechanics in conjunction with observation of abnormal local or global structural, adaptive, and/or tonal (both muscle and autonomic) tests, the latter of which reflects abnormality within the complex interaction between local/peripheral state and central command control [181-183]. Location, analysis, and correction of VS via the specific chiropractic adjustment (a mode of care termed “LACVS”) are intended to restore structural, biomechanical, and neural integrity to the spine, with an overriding intention of facilitating neurally modulated adaptive function in the body. Underscoring the distinctions between these modes of care, recent research indicates that an adjustive thrust targeted at an upper cervical vertebral segment bearing markers of VS elicits a distinct change in neural/sensorimotor parameters, via N30 complex amplitude, while an adjustive thrust delivered to a “random” vertebral level with no evidence of vertebral dysfunction (a “manipulable lesion”) fails to elicit a neurophysiological change [184].

These methodological and outcomes distinctions between these two modes of chiropractic care, spinal manipulation for treatment of pain and improvement of mobility as compared to LACVS for restoration of normal neurally mediated adaptive function, become an important consideration for planning and executing studies that explore potential multiple outcomes of chiropractic care. The practical choice of whether to assess study subjects for VS per level comprises an additional, paramount distinction between these two modes of chiropractic intervention, and it exposes a possible methodological flaw in studies that fail to categorize subjects according to VS status. Specifically, by its nature, any study designed to assess the potential impact of spinal adjustive intervention on NEI modulators or outcomes presupposes the possibility of a spinal clinical abnormality (such as VS) that could impact global neurally mediated NEI adaptive functions and outcomes. The reproducible observations and reports within our compiled database of NEI responses via correction or reduction of VS, as compared to general spinal manipulation, support this hypothesis. To clearly distinguish spinal manipulation (for manipulable lesions) from specific chiropractic adjustment (for VS), a secondary, operational definition of VS can be helpful in that it converts the primary conceptual definition of this entity into a practical method of ascertaining its presence and assessing changes in its severity, in temporal relationship to the adjustment [181,185,186]. If a given observed maladaptive syndrome is a collection of signs and symptoms caused by VS, then ultimately, supportive evidence for the involvement of the clinical entity of VS is provided by the successful application of its correlate solution, the chiropractic adjustment, with temporally correlated resolution or reduction of the manifesting maladaptive signs and/or symptoms. The presence of VS could therefore be understood by the reliable, predictable, temporally correlated reduction or resolution of its quantifiable manifesting structural and functional maladaptations after delivery of the chiropractic adjustment, as compared to placebo control, as follows. Biomechanical and structural maladaptive manifestations of VS are quantifiable by multiple means [187], including weight-bearing radiography [188], posture and gait analysis [189,190], balance testing and joint repositioning [191-193], and range of motion or static/motion palpation [187,194]. Neural/neurophysiological maladaptive manifestations of VS can be observed by paraspinal thermography and sEMG [195], HRV [196], blood pressure assessment [46], tonal/tissue changes, and functional leg length analysis [197-199]. In everyday clinical practice, whether using a postural, segmental, or tonal approach, the presence and subsequent correction of VS can be reliably assessed via a multidimensional, combinatorial battery of such tests as listed above [182]; selection of outcomes assessments is dependent on the nature of the model of practice employed by the practitioner, with correction/reduction of VS as a common denominator. By comparison, the criteria for spinal manipulation therapy include pain/tenderness upon palpation, reduced range of motion, or spinal stiffness [200], therefore using an operational definition focused on pain and/or range of motion. Considering the criteria described above, it becomes clear that the clinical targets of spinal manipulation therapy and the outcomes it elicits are easily distinguishable from the clinical intentions and outcomes of location, analysis, and correction of VS via the specific chiropractic adjustment.

From a research methods standpoint, then, if VS is a causative agent of NEI dysfunction (a compendium of maladaptative syndromes), it becomes necessary to categorize study subjects for NEI outcomes studies (including symptomatic, or “healthy” asymptomatic subjects) according to presence or absence of identifiable VS, per level, in order to establish appropriate experimental and sham/control arms. If VS is a causative agent for NEI dysfunction/maladaptation, then studying spinal interventions that are performed on patients lacking this putative causative agent (no VS present) would likely lead to null/negative outcomes, and therefore to inappropriate confirmation of the null hypothesis, a classic methodological flaw. Conversely, perhaps NEI or other salutogenic changes observed after spinal manipulation may in fact be bona fide, particularly if (uncharacterized) VS was unknowingly present at the level(s) manipulated, much like one can hit a target on a bullseye if using birdshot.

While a chiropractic adjustment-centered approach acknowledges VS as a variable important to trial design and outcomes, spinal manipulation by its nature does not assess for VS. This discrepancy raises concerns regarding the interpretation of control and experimental outcomes of trials utilizing general spinal manipulation as an interventional approach, particularly those reporting negative results: even if both modes of chiropractic care may address the same spinal segment, perhaps it is the functional condition of the segment and its correction, rather than the segment per se, that dictates outcomes. The next horizon will be to distinguish in more detail the effects of general mobilization or spinal manipulation versus the specific chiropractic adjustment, per level; for example, comparing interventions to a spinal segment that does versus does not bear VS as measured by validated tests [182].

Bearing this distinction in mind, in the following summary sections the general term “chiropractic care” is intended to encompass the more general model of care inclusive of both spinal manipulation and traditional chiropractic care, but if the specific designation “chiropractic adjustment” is used, specific assessment for and correction of VS as a clinical variable was deployed, and results will be discussed accordingly. Importantly, given the clarifications described above, in future published chiropractic studies selection and application of the terms “chiropractic adjustment” or “spinal manipulation” should follow the criteria set forth above, to accurately delineate the functional difference between these two modes of spinal care for research purposes. Deploying the two terms accurately will allow this field to correct the past inappropriate conflation of these non-interchangeable terms [186], leading to a more discrete understanding of the contribution of the functional status of the vertebral level to outcomes of chiropractic care.

The following 10 sections explore the references identified that met our inclusion criteria per keyword, with a consideration of strengths, limitations, biological plausibility, and future directions for each area of study. The inclusion criteria for this integrative review were purposefully chosen to create a bias toward research documenting quantitative results and should be interpreted as such. Reports describing negative results will, however, be discussed within each results section, to add context. While the theoretical and practical neurophysiological mechanisms by which spinal manipulation [117,201,202] or chiropractic adjustment delivered for VS [181,203,204] exert local and global effects on the nervous system have been reviewed, biologically plausible mechanisms by which chiropractic care may exert effects on the integrated NEI system and its components specifically will be discussed per keyword. Two keyword searches (“Autoimmune” and “Transforming Growth Factor/TGF”) did not yield articles published as of 2022 that met our inclusion criteria, and therefore these topics will not be addressed here.

Blood Pressure

Blood pressure is an integrative adaptive response that weighs inputs from each of the NEI supersystems [205-207]. Restoration of normal blood pressure can be considered not simply as a neural or endocrine change, but rather as a coordinated action, since both short-term and long-term blood pressure regulation requires the integrated actions of multiple cardiovascular, renal, neural, endocrine, immune, and local tissue control systems [208,209]. High blood pressure is connected to multiple worsened patient outcomes; every 10mm mercury reduction of hypertension significantly reduces the risk of coronary heart disease, stroke, and heart failure, and leads to a significant reduction in all-cause mortality [210]. Restoration of a normotensive state from a maladaptive state of either hypertension or hypotension can be considered a salutogenic change, since this would represent a positive change on the health ease/disease continuum [28,29]. Available research literature describing the effect of chiropractic care on blood pressure contains conflicting reports, likely due to methodological differences, and in sum these data support a role for restoration of normal blood pressure with chiropractic care, particularly with correction or reduction of VS in the upper cervical spinal region.

The collection of 30 references identified in this search [46-75] contains 10 qualitative case studies with quantitative assessment of changes in blood pressure, 14 trials (observational, cohort, RCT), and six reviews demonstrating the impact of chiropractic care on blood pressure. Of the identified reviews, four report “mixed results” yet conclude that chiropractic care yields promising positive change in individuals with hypertension [50,51,55,64] while one review notes changes in systolic blood pressure but expresses concern regarding potential sources of bias in trial design [58], a conclusion since challenged via a more recent analysis [50]. Interestingly, of the 14 identified trials reporting positive change in blood pressure, eight utilized assessment and adjusting techniques for the location and correction of VS [46,53,56,59,60,65,66,70], and eight employed one-variable, upper cervical trial designs [46,53,56,60,65,66,69,74], suggesting that upper cervical VS may be important for chiropractic care-mediated modulation of blood pressure. Consistent with these data, nine case studies identified in the search show a similar trend: each of these references employed techniques to assess for and correct VS [48,52,54,61,62,67,68,71,72,75], and six of these utilized specific upper cervical approaches [48,52,54,62,71,72], though interpretation of case studies as a group must be approached with caution due to risk of bias intrinsic to case studies. It is essential to note that the demographic characteristics of study groups are not consistent between many of these studies, complicating their consideration as a group: for instance, while several studies include only unmedicated participants with stage I hypertension [46,47,49,69], other studies include normotensive individuals and/or do not offer details regarding medication use [53,56,59,63,65,66,70,73,74]. Importantly, seven negative/null studies not meeting our inclusion criteria were also identified in the process of our literature search, each with methodological flaws that may complicate the interpretation of their results and may have resulted in false confirmation of the null hypothesis. Three of seven negative studies utilized methods that may have been insufficient to test the hypothesis, via the use of normotensive rather than hypertensive study participants [118,211,212]. Six of seven negative studies employed a full-spine intervention approach or were unclear regarding which specific vertebral levels were addressed, and therefore these approaches may not have impacted segment(s) that may impact blood pressure, particularly if functional perturbation (VS) at any specific level is indeed implicated [118,211-215]. Five of seven did not assess manipulated spinal segments for VS as a clinical variable that may impact blood pressure [118,211-213,215], a choice that may have complicated their trial design and outcomes. The remaining study of the seven identified that reported no change in blood pressure with the chiropractic adjustment successfully utilized a specific upper cervical analysis and adjusting approach for VS at a single vertebral segment (C1) [216], yet its conclusions and comparability to other studies of unmedicated subjects that yielded a quantitative change in blood pressure may be complicated by the investigators' choice to study participants continuing to take medication to modulate blood pressure. This methodological flaw may have led to incorrect confirmation of the null hypothesis, since potential blood pressure responses may be affected, blunted, or negated by medications designed to modulate hypertension [216]. The possibility that publication bias may have prevented dissemination of well-designed studies that do confirm the null hypothesis must also be considered; however, in sum, an in-depth examination of available research that includes consideration of VS does support a theory that upper-cervical-specific approaches to analysis and adjustment may correct or reduce VS and thereby impact blood pressure, whereas an approach of full-spine manipulation without assessment for VS may yield the “mixed results” reported in reviews and discussed herein, depending on whether a vertebral segment (upper cervical or otherwise) affected by subluxation was present, addressed, and was affecting blood pressure. It is also important to consider that within this theory, VS at one segment or area may be most commonly associated with this abnormal physiology and yet maladaptive blood pressure or any other abnormal physiology that could either directly or indirectly impact blood pressure could also be evoked by VS at different segments. Considering all of the above factors together may therefore explain why currently available meta-analyses conclude the evidence is “mixed, but promising.”

Of the papers identified, Bakris et al. [46] is a particularly striking study due to its trial design (double-blinded, placebo-controlled), its clinical specificity (chiropractic adjustment was constrained to a single subluxated vertebral segment); both experimental and sham groups were confirmed to have VS at the same spinal level, C1, per National Upper Cervical Chiropractic Association (NUCCA) protocol [217]; both pre-adjustment and post-adjustment assessments for VS were employed, including radiography and tonal leg/postural assessments), and, importantly, the sham control was designed to be indistinguishable to the patient from an authentic alignment, thereby reducing the potential for placebo effect. In this study, both hypertension and rotational/lateral atlas displacement improved in adjusted patients but not sham-treated individuals, both immediately following adjustment and in a sustained manner over a course of eight weeks, strongly supporting a connection between correction of C1 VS and improvement or resolution of hypertension. These results were both statistically and clinically significant, thereby rejecting the null hypothesis. It is of interest to compare and contrast this study with the aforementioned 2016 Goertz study [216], which was ostensibly designed to confirm or refute Bakris’ findings. This study, while it used a similar adjustment approach (upper cervical, assessing and adjusting C1 for VS), can be distinguished from the Bakris study by an important difference in the respective study populations: while participants in the 2007 Bakris study were “washed out” of medications, participants in the 2016 Goertz study remained on hypertensive medications. Echoing concerns highlighted in the previous paragraph, this choice comprises an important methodological distinction between these two studies that renders them non-comparable. The conclusions of the Goertz study, no change in hypertension observed after adjustment, will naturally only apply to individuals taking medication, whereas the conclusions of the Bakris study, normalization of blood pressure after a chiropractic adjustment, suggest that the hemodynamic homeostasis of stage I hypertensive individuals not taking medication for their condition may benefit from chiropractic care.

Given that several reviews have described “conflicting evidence” regarding whether chiropractic care modulates hypertension, one must consider these mixed data bearing in mind distinctions in the research methods of the individual reports assessed in these reviews, including (1) studies that test one variable (adjustment of one vertebral level) cannot be directly compared to studies that contain multiple variables (adjustment of multiple vertebral levels, left to the “discretion of the practitioner”); (2) assessment of intervention-related blood pressure changes in normotensive individuals cannot be compared to assessment of subjects with abnormal blood pressure; (3) the use of a different patient population (medicated, as compared to not medicated) renders studies non-comparable; and (4) studies that do and do not test for VS also cannot be considered together, as VS itself may present a physiological variable requiring accommodation in research methods design. A more in-depth appraisal of all available studies is beyond the scope of the present paper, however, we highlight here the presence of research methods concerns that may have impacted previous analyses and subsequent systematic and meta-analyses. We therefore advocate for an updated and rigorous meta-analysis of available data with critical appraisal of all relevant studies, including a delineation of whether VS was assessed, and that controls for confounding factors such as medication use and lifestyle factors that can influence the outcomes of such studies. Future research on this subject should include a variety of study designs that clearly delineate the demographic characteristics of study participants (specifically, whether the study subjects are normotensive, hypo- or hypertensive, and whether included participants are taking medication that may affect blood pressure) and should address the multifaceted nature of blood pressure regulation.

Multiple overlapping pathways by which VS, particularly at the upper cervical spine, may exert blood pressure effects have been suggested and explored. Both somatic and autonomic influences have been suggested [55,218], and these possibilities are not mutually exclusive. For example, extrapolating data from laboratory studies [219,220], Torns [65,66] elegantly suggests a pathway that may mediate reflex changes in autonomic variables after neck muscle spindle afferent activation: the nucleus tractus solitarius (NTS, a site of cardiorespiratory regulation that receives not only visceral, but also somatic, afferents [221]) and intermedius nucleus of the medulla (InM) receive information from upper cervical proprioceptive afferents via the recurrent meningeal nerve and dorsal root ganglion, and/or the nodose and jugular ganglion. In Torns’ model, mechanical pressure from atlas displacement may alter proprioceptive neural input from upper cervical somatic afferents to the NTS, which may, in turn, inform and influence integrative adaptive control of blood pressure via the cardiac autonomic network (CAN) and its efferent [222]. Interestingly, such upper cervical proprioceptive afferent input to NTS may modulate either hypo- or hypertension, as these individual circuits in NTS can be selectively activated [223], consistent with Torns’ observation of normalization of hypo as well as hypertension after upper cervical adjustment [66]. Alternatively, VS at other spinal levels (or multiple spinal levels, in a summation model) may elicit adaptive change in blood pressure as a clinical outcome via direct mechanisms other than upper cervical, including modulation of the pressor reflex, muscle sympathetic nerve activity (MSNA) as mediated by somatosensory afferents, effects on baroreceptor compliance, or modulation of cervicosympathetic reflex or other neural networks [56,64], or via coordinated systemic effects resulting from flux within the NEI supersystem, as mediated by changes in cortisol [224] or inflammation [209]. It is interesting to consider, as well, the effects that medication(s) may have on any of these putative pathways, as each category of blood pressure medication would be expected to have impacts on cardiovascular hemodynamics [225], underscoring the importance of a trial design that excludes participant medication use. Further, in-depth exploration will be necessary to fully study a potential upper cervical etiology of hypertension, and to elucidate other multiple, overlapping, or layered biological mechanisms by which VS and the specific chiropractic adjustment can alter the dynamic equilibrium of blood pressure.

Cortisol

The small molecule cortisol, a glucocorticoid steroid hormone and classical neuroendocrine modulator, is often referred to as a “stress biomarker.” Cortisol’s diverse actions include mediation of the stress response, regulation of metabolism, and modulation of the inflammatory response and the immune response [226]. Pathological dysregulation of cortisol levels may result in outright diseases such as Addison’s disease or Cushing’s syndrome; however, sub-pathologic dysregulation can also result in numerous health presentations including but not limited to cardiovascular disease, autonomic dysfunction, metabolic syndrome/insulin resistance, cognitive decline, fatigue/depression, and dysregulated inflammation/autoimmunity [227]. The flux of cortisol within a normal range (either increase or decrease) reflects both the body’s normal dynamic equilibrium and its adaptive capacity [226]; any intervention that impacts cortisol flux may therefore impact adaptive responses as executed by the neuroendocrine supersystem. Improvement of cortisol regulation within the body would represent a salutogenic change [28,29], via enhancing or restoring the body's intrinsic ability to shift metabolism, inflammation, and autonomic state in response to stress.

The present literature search yielded 11 references reporting alteration of cortisol levels after chiropractic intervention [76-86]: three case studies demonstrating a quantitative change in salivary cortisol [76-78], and eight-level V or higher [38] references including five clinical trials [79-83], and three reviews [84-86] quantifying change in cortisol with chiropractic care. The latter category includes two systematic reviews/meta-analyses, one finding moderate quality evidence that spinal manipulation may influence cortisol levels post-intervention [86], and a more recent work finding “mixed effects” of chiropractic care on salivary/serum cortisol levels in people with spinal pain, a discrepancy the authors suggest may be due to differences in study populations [84]. This finding of “mixed results” brings forth an important point: as cortisol normally crests and falls with circadian rhythm, perhaps absolute change as compared to baseline rather than the specific direction of change should be considered, or alternatively, results may be subdivided into “increase” and “decrease” categories for the purpose of meta-analyses. Accordingly, the identified level V or greater reports include a controlled, repeated-measures, single-blind randomized study showing statistically significant changes in serum cortisol in adjusted patients as compared to sham [81], a controlled laboratory study finding that thoracic spinal adjustment resulted in an immediate decrease in salivary cortisol concentration and reduced testosterone: cortisol ratio six hours after intervention [82], as well as an RCT of 83 chronic mechanical neck pain patients in which an increase in salivary cortisol concentration was identified immediately following chiropractic care as compared to control [83]. Of note, one null study not meeting our inclusion criteria for the final database was restricted to asymptomatic subjects and did not show change in salivary cortisol in spinal manipulative therapy (SMT) as compared to sham [228] underscoring the possibility that an effect on cortisol by spinal adjustment may not be universal, but rather context-specific (e.g., significant induction of change in cortisol levels may not be readily observed in normal patients, but may be more salient in patients with abnormal cortisol flux, response, or levels, such as may be caused by segment-specific VS that may cause varying degrees of dysafferentation or dysautonomia [181]), necessitating further exploration. Taken together, the above data provide foundational preliminary evidence that cortisol levels can be impacted by chiropractic care and therefore could impact NEI outcomes such as blood pressure regulation [224] and hormonal, cognitive, metabolic, and immune function [229], including modulation of inflammation [230,231].

These early data support a model in which chiropractic care may affect the flux of cortisol within the body’s homeodynamic system, potentially in a context-specific and/or bidirectional manner. A clear biological pathway by which VS and chiropractic adjustment may affect cortisol flux has not yet been elucidated, though both autonomic effects and HPA-axis effects present possible etiologies. In a recent review, chiropractic care was proposed to modulate the autonomic nervous system (ANS) through activation of the parasympathetic nervous system, reduction of sympathetic nervous system activity, and synthesis of neuroendocrine factors, impacting major depressive disorder [232]; modulation of the ANS could also exert effects on cortisol levels and flux, via actions on the adrenal gland [233]. Alternatively, recent data suggest that the HPA axis regulation system interacts with the somatosensory system [234], bringing forth the possibility that altered somatosensory input via VS may impact hypothalamic-pituitary-adrenal axis flux and release of CRF, corticotropin-releasing factor, impacting cortisol levels and flux [222,235]. Consistent with this possibility, pain/nociception has been shown to modulate both HPA axis sensitivity and cortisol release [236]. An important limitation of this collection of articles is that these data have not yet directly addressed whether these observed changes in cortisol result in clinically significant patient outcomes. As such, future studies (including longitudinal) of changes in cortisol after chiropractic adjustment as have been proposed [237] should be directed towards exploring changes in cortisol in various patient populations after specific adjustments per level(s) bearing indicators of VS, and incorporating clinical endpoints that reflect cortisol-related pathologies as listed above, as well as research directed towards elucidating precise neurobiological pathways by which spinal adjustment can affect cortisol levels.

Cytokine

The term “cytokine” encompasses a broad family of extracellular proteinaceous small-molecule NEI regulators that have diverse and complex effects on inflammation/immunity [238], neuromodulatory effects [239], and regulatory effects on endocrine function [240]. Cytokines influence cell, tissue, and system homeodynamics through both transient and longer-lasting alterations [241] and therefore the term “cytokine” was selected as a candidate indicator for global homeodynamic functional alterations that may result from spinal adjustment. One clear biological pathway by which chiropractic care may elicit alterations of cytokines (or any small molecule messenger, including interferons (IFNs), interleukins, TNFa, and substance P) is mechanical deformation, which can instigate the release of small molecule messengers from either affected cells or nearby extracellular matrix and fascia [242-244]. Twelve references discussing the impact of chiropractic intervention on cytokines were identified in our search [84,85,87-96]. As “cytokine” is an umbrella term that covers several other keywords used in this search, these identified references will be considered in general in this paragraph and in more detail in their respective sections below, to emphasize their respective unique functional characteristics and/or distribution and/or compartmentalization within the NEI supersystem. This collection of articles can be divided into three reviews [84,85,96] and eight trials, including a collection of RCTs exploring short-term in vitro cytokine biomarker responses in cells from individuals undergoing SMT [91-95] and several RCTs detailing changes in cytokines in individuals undergoing SMT, as measured by direct lab testing [87,88,90]. The subject of assessment of changes in cytokines with spinal adjustment has garnered much interest in the past two decades, and the early evidence described herein supports the possibility of local or global change of cytokine biomarkers as a result of SMT or chiropractic adjustment. However, it is important to consider that this field of study is in its relative infancy, since such RCTs are challenging to plan, fund, and execute. One noted limitation intrinsic to the youth of this field of study is a lack of demonstrated, direct clinical correlation between changes in cytokine biomarker levels and impact on patient outcomes, resulting in as-yet unclarified clinical significance [127]. Future directions correlating changes in cytokine flux with clinical outcomes of spinal adjustment will likely be the next step as this field of study matures, and may include studies that would allow for stronger conclusions regarding the connection between spinal adjustment and cytokine-mediated physiological changes. Ongoing studies exploring relationships between chiropractic care and serum cytokines may also benefit from updated technology such as multiplex testing, through which coordinated changes in multiple small molecule messengers such as interleukins, tumor necrosis factor (TNF), and IFNs can be assessed over time. Design of studies that compare serum samples from each subject to their own baseline, individually rather than as a cohort for trials, may be helpful since levels of cytokines may differ among individuals [245].

Heart Rate Variability (HRV)

In order to efficiently adapt to its internal and external environment, the body must be capable of quickly modulating any of its activities. The capacity to rapidly change parameters within a normal physiological range in response to any perturbation is a foundational and essential element for the body’s entire range of processes, from systemic/whole-body to cellular adaptability. Any deficit or obstruction to the ability to adapt would be expected to detract from the body’s self-regulatory capacity, resilience, and ultimate survival [1]. HRV has long been considered one of the most promising biomarkers of autonomic activity [246], though controversy exists as to whether it comprises a stand-alone biomarker with specific diagnostic or predictive power, given its complex nature as reflecting the summary of multiple physical and psychological stressors and affected by medication use, age, physical fitness, cardiac pathology, and other factors [247]. It is considered by some a surrogate parameter of the complex interaction between the brain and cardiovascular system that reflects both adaptability and robustness within the cardioautonomic system [248] and to be a proxy indicator of neurovisceral integration (NVI) and NEI adaptability [249]. HRV consists of changes in the time intervals between consecutive heartbeats called interbeat intervals (IBIs). Rather than having a metronomic rhythm, the beating of the heart exhibits micro-variations in IBIs, a variability that is quantified via metrics such as “time domain,” “frequency domain,” “non-linear” metrics, and “coherence" [250-252]. Abnormally low HRV is a strong, independent predictor of future health problems including worsened cardiovascular disease outcomes [253], inflammatory and immune [254,255] outcomes, and is associated with all-cause mortality [253]. Reduced HRV is also observed in patients with autonomic dysfunction, including anxiety, depression, asthma, and sudden infant death [252]. Conversely, higher HRV is used to predict fatigue, recovery, and performance in athletes [256] and is a salutogenic marker predictive of enhanced stress resilience and cognitive performance [257], healthy longevity [258], and cancer survival [259]. HRV may therefore be viewed as an integrative measure of systems biology [248,249,252], that both considers input from and influences immune, metabolic, endocrine, and respiratory processes. Enhancements in cardio-autonomic resilience and adaptability as reflected by HRV would represent positive change on the health ease/disease continuum [28,29], comprising an observable manifestation of salutogenesis.

Twenty-nine references were identified in our search that assess the relationship between chiropractic care and HRV [63,64,70,73,97-121]. The available literature can be grouped into 12 case studies or case series reports [100,102-105,107-110,113,120,121], 12 cohort, RCT or other level V studies [63,70,73,98,99,101,111,112,115,116,118,119], and five reviews [64,97,106,114,117]. This collection of research papers is heterogeneous by nature, given the permutations of HRV metrics, differences in measurement conditions (including sampling duration, patient position, and breathing instructions), and data management, particularly when considered in the context of the many diverse approaches to chiropractic care. The five identified reviews generally agree that chiropractic care exerts an influence on the ANS as measured by both time and frequency domain metrics of HRV. Of note, the HRV field has some disagreement as to whether frequency domain metrics (low frequency (LF) and high frequency (HF)) are universally accepted as generalizable to specific aspects of autonomic function, such as sympathetic or parasympathetic activity, and whether frequency domain metrics can be easily and reproducibly measured pre- versus post-adjustment [250,260,261]. As such, conclusions of studies describing changes in frequency domain parameters should be considered with caution. Therefore, we have chosen to narrow our focus for the purposes of the current literature review to reports that utilize time domain metrics (rMSSD (root mean square of successive differences), SDNN (standard deviation of NN intervals), and LnRMSSD (natural logarithm of rMSSD)), which are mathematically simpler and less affected by measurement conditions or data management strategies [63,73,104,105,116,118,119]. Of these works, Zhang et al. [119] are notable in that both immediate and sustained improvements in SDNN were observed in the 539 patient cohort after chiropractic adjustment delivered to correct VS, as compared to baseline HRV. While this study lacked a cohort unadjusted control, the observation of an increase of time domain metrics with chiropractic care has since been repeated by several studies including three controlled RCTs [63,73,104,105,116,118], supporting Zhang’s contention that chiropractic care enhances HRV. Considered together, this limited but promising collection of studies suggests a salutogenic role for chiropractic adjustment of VS, via enhancement of adaptability and resilience as reflected by HRV.

Our literature search also identified two studies not meeting inclusion criteria that reported no change in time domain metrics with chiropractic care [212,262], discussed here for comparison and context. Two important differences can be noted between these negative studies and the seven studies that report the change in time-domain HRV with chiropractic care: while the seven studies described above reporting quantitative change in HRV included symptomatic patients with presumably abnormal HRV values pre-intervention, the two null studies assessed the effect of spinal manipulation only on asymptomatic subjects whose HRV values would be expected to flux within normal parameters. It is also possible that HRV would only be affected within a certain patient cohort, with as yet undefined specific characteristics. Further, these studies addressed different spinal regions (cervical/lumbar versus thoracic, respectively) raising the possibility that no effect was observed in the null studies due to incorrect selection of vertebral level intervention and/or inadequate characterization of subjects’ VS functional status. Additionally, echoing the previous discussion of the methodological concerns within the research literature on blood pressure and chiropractic care, repeated observation of change of HRV with spinal adjustment supports the likelihood of a spinal abnormality (e.g., VS) that affects HRV. This possibility underscores the necessity of reconsidering the use of “asymptomatic patients” and/or adjusting per level without assessing for VS: if VS were causative of HRV functional abnormality, then its presence would be expected to be requisite to yield HRV outcomes with spinal adjustment, and no changes in HRV would be expected with adjustment in the absence of VS at that same vertebral level. We hypothesize that the selection of the appropriate vertebral level for a chiropractic adjustment is important for observing changes in cardioautonomic function as measured by HRV and that failure to select the appropriate vertebral level would constitute a confounding error in research methods that would dictate observed outcomes. Future research on HRV should include trials that randomize patients to receive adjustments for VS vs. random selection of vertebral level.

The available evidence suggests the utility of chiropractic care for the improvement or restoration of normal HRV as measured by time domain metrics. Future HRV research in the chiropractic field should bear in mind the many technical challenges with quantifying HRV that need to be appreciated and addressed [263]. Ongoing research should be planned with consideration of standardization of approaches to promote comparability of results such as utilizing research-validated data collection and management approaches [264] including a collection of HRV data via ECG rather than plethysmograph; "cleaning" of data with software that detects and removes artifacts such as ectopic beats or missed data; employing a standardized five minute sampling time at a defined time of day, with patients positioned seated and breathing normally at between 11 and 20 respirations per minute; including the metrics SDNN and rMSSD such that data may be compared to known normative standards; and publishing tables of all HRV data obtained in studies [250]. Researchers should pay particular attention to HRV terminology and discrete limitations therein since each individual HRV metric represents one unique facet of HRV and these are specific, not interchangeable, and not generalizable. For instance, the time-domain metrics SDNN and rMSSD are distinct: while both SNS and PNS activity contribute to SDNN, making this a good overall descriptor of autonomic activity, rMSSD is the primary time-domain measure used to estimate the vagally mediated changes reflected in HRV [250]. Each of these metrics is also limited: SDNN conveys no information regarding autonomic balance (SNS/PNS) per se, and is important to discern that rMSSD is not a direct measurement of vagal tone as a generality, but rather a reflection of vagal modulation of heart rhythm. These metrics and their definitions should be employed precisely within ongoing HRV research within the chiropractic field, to best interface it with HRV research in other fields.

The observed change in HRV with chiropractic care indicates modulation of autonomic activity, which unto itself comprises an effector arm of the NEI supersystem [10,265]. The precise neuroanatomical means by which chiropractic care, and VS, may exert neuromodulatory effects on HRV have yet to be elucidated and may include indirect effects via modulation of sympatho-excitatory response [55] or alteration of somatosensory input to the CNS from the upper cervical spine [220], or perhaps via modulation of vagal (SNS/PNS) input within the hierarchy of Thayer’s NVI model [249]. Interestingly, “innocuous stimulation” to the upper cervical spine evokes HRV changes [98], and osteopathic suboccipital decompression enhances HRV indices in healthy subjects [266,267] supporting the hypothesis that somatosensory efferent input from the upper cervical spine, or dysfunction thereof via VS, may influence HRV. The emerging field of research exploring the impact of chiropractic care on HRV contains both clues and opportunities for the broader-reaching effects of chiropractic care to be discovered and understood.

Immunity/Immune System

The general term immunity, inclusive of innate immunity, adaptive immunity, and passive immunity, encompasses an expansive swath of biological function. In an integrative systems view of neuroendocrine immunology, immune function represents one leg of a three-legged stool: any impact on the nervous or endocrine system would be expected to affect the immune system, and vice versa, with a continuum from subtle to profound potential impacts on physiological and clinical outcomes [20,265]. The means by which immune functions may be modulated by the nervous and/or endocrine system are multiple and overlapping. These include direct innervation of immune organs, sharing of messenger molecules and receptors, sites of physical overlap such as found within the glymphatic and CNS lymphatic systems, and both direct and indirect reciprocal communication between the endocrine axis, the ANS, and the immune system. Furthermore, NEI cross-modulation is likely to be influenced by, and influence the microbiome via the microbiota-gut-brain (MGB) axis [268]. The complex interactions of the nervous, endocrine, and immune systems orchestrate the symphony that is an adaptive allostatic response, affecting not only immune function but also multiple physiological processes including stress responses, growth and development, reproduction, metabolism, and circadian rhythms. “Immunity,” per se, can therefore be viewed as a central cog in the greater machine of integrated physiology. While diminishment of immune function renders the body susceptible to disease and dysfunction, enhancement or restoration of any aspect of function of the immune system would be considered salutogenic by definition [28,29] as such improvement would be expected to create a positive change in the health ease/disease continuum.

The broad question of whether chiropractic care impacts immunity is challenging to consider simply, and as such the aim of this “immune/immunity” keyword search summary is to describe existing evidence that chiropractic care impacts immune function as an entity on multiple levels, from basic science exploratory studies to clinically-relevant outcomes. As with the keyword search term “cytokine” above, other unique keyword searches that directly relate to “immunity” such as interleukin and interferon are considered separately in more individual detail in the following sections. Twenty-seven references were identified in our search describing the relationship between chiropractic care and various aspects of immune function: 12 case studies, five research trials, and 10 reviews [85,93,96,122-145]. Of these original research studies meeting our inclusion/exclusion criteria, notable findings include one early set of pilot studies that report trends towards shifts in blood markers/CBCs in adjusted subjects [124,125], and another trial that reports lymphocytic activation in adjusted subjects as compared to control [132]. Similarly, another trial details an increase in antibody secretion from cells isolated from subjects undergoing spinal manipulation, as compared to sham control [93]. The present search also identified a number of case studies with quantitative evidence of functional improvement of autoimmune and infectious processes, such as the resolution of chronic and/or treatment-recalcitrant idiopathic arthritis, asthma, otitis media, and myasthenia gravis following the introduction of chiropractic care [123,128,131,134,136,140,143], and/or report of reduced frequency of illness or school misses after commencement of chiropractic care [133,142,144,145,152]. Together, these original data are supportive of chiropractic care impacting immune adaptability in an evident model of practice.

Several reviews identified in our search discuss the available literature (including research published before 2000, excluded from the present search) and explore biologically plausible mechanisms by which chiropractic care may enhance immune function by triggering activation of the neuroendocrine system. Three early (pre-2010) narrative literature reviews [126,129,139] discuss a small but growing body of animal and basic science research exploring the potential impacts of chiropractic care on the immune system, via neuroimmune cross-communication. One review contends that while these early studies do not directly examine the effect of chiropractic care on immune functional outcomes, the available basic science studies do give broad mechanistic support for and are suggestive of overlapping mechanisms by which spinal influences may mediate a clinically significant impact on immune function, thereby providing justification for additional studies [126]. Some of these early studies present conflicting data, for which potential contextual explanations have been outlined including research methods considerations including the timing of data acquisition [96,130]. Indeed, one critical review that we identified in our search gives consideration to similar research methods issues, including small sample size, heterogeneity related to methods of biomarkers collection and sham procedures, and lack of studies on symptomatic subjects. These issues preclude any understanding of how the elements of innate and adaptive immunity would function after SMT under pathological circumstances, in a clinically relevant manner [130]. Similar concerns were echoed in a narrow-scope systematic review of eight studies two years later [127]. Most recently, a broader-scope narrative review by Haavik et al. [135] offers that 18 of 23 studies considered show a significant effect of chiropractic care on neuroimmune markers. The authors outline multiple neurologically based, research-supported mechanisms by which chiropractic care may elicit this response. It is of note that most of these early exploratory studies that utilize spinal manipulation as an intervention approach fail to assess VS status, segmental or global, in either sham or experimental arm, thus creating a potential research methodological error that would further complicate the interpretation of results. Therefore, early research on the relationship of chiropractic care to immune function offers promising observations that create opportunities for further investigation using more refined methods, including the use of VS and sham cohorts, and the inclusion of measurement of clinical endpoint outcomes.

Some controversy regarding the application and utility of chiropractic care for the enhancement or restoration of immune adaptability has arisen in recent years. The coronavirus pandemic of 2020 brought a flurry of debate surrounding the relationship of chiropractic adjustment to the function of the immune system. The World Federation of Chiropractic (WFC) promoted a self-published, non-peer-reviewed “rapid review” [269] of the potential effects of spinal adjustment on immune parameters that discussed only six pre-selected research papers and a “meme” that was circulating on social media, therefore raising questions regarding bias and flawed research methods. It is interesting that the narrow scope of this ”white paper,” not inclusive of all available literature by virtue of selective exclusion criteria, is echoed subsequently in a narrow-scope review [127] whose corresponding author has affiliations with the WFC, including the authors of the “rapid review,” and who co-authored a “unified statement” signed 140+ chiropractors, calling “on regulatory authorities and professional leaders to take robust political and regulatory action against those claiming that chiropractic adjustments have a clinical impact on the immune system" [270]. Of note, with roughly 100,000 chiropractors worldwide [271], these 140+ signatories comprise 0.14% of chiropractors, rather than a "unified statement" as claimed. Through these documents, the WFC has promoted conclusions and short-sighted advice including the contention that chiropractors worldwide should refrain from statements regarding the utility of chiropractic care for immune health and adaptability. These myopic claims have since been challenged by more recent and in-depth reviews of research literature on this subject [85,137]. In addition, a Best Practice/Practice Guidelines overview of 125 research papers related to the impact of chiropractic care on immunity has been issued. This document, peer-reviewed by 200 chiropractors worldwide, provides an evidence base for field practitioners [138] and clearly maps out the foundational elements and blueprints for the planning and execution of future research on the impact of chiropractic care on immune function. To overcome the present controversy, all current evidence and reviews should be considered, in conjunction with the Foundation’s Best Practices/Practice Guidelines document and in an EIP context, by any entity advising chiropractic boards and governing agencies that the chiropractic scope of practice should exclude immune adaptability.

Most recently, Schalow et al. [141] published results from a translational research-centered observational trial reporting the effect of specific chiropractic adjustment on levels of secretory IgA (SIgA) in chiropractic patients, a study that begins to bridge the gap between exploratory basic science and clinically relevant outcomes-based chiropractic research on immunity. The report, “Secretory Immunoglobulin A and Upper Cervical Chiropractic: A Preliminary Prospective, Multicenter, Observational Study,” details observations gathered from n=41 upper cervical chiropractic patients under the care of five qualified diplomates or fellows (DCCJP, FCCJP) specializing in UCAT (Upper Cervical Adjusting Techniques), whose analysis and adjusting approach is specific for location and correction of VS at C1/Atlas. The strength of the research method employed for this study, choosing to limit their study cohort to VS at one variable (C1), parallels Bakris’ well-designed clinical trial on the effect of chiropractic adjustment at C1 on hypertension [46]: in both cases, limiting experimental variables allows for simpler interpretation of data. SIgA, an indicator of systemic immunity, is an integral part of the body’s “first line of defense” [272]. An accessible biomarker of mucosal immunity, SIgA provides a useful indicator of several key parameters including individual and community immune response, disease severity, and clinical risk [273]. Lower levels of SIgA are correlated with infection risk [274,275] and recent studies have suggested that salivary IgA seems to be an effective outcome for controlling the risk of developing URTIs in athletes [276]. As such, any intervention that enhances SIgA levels would be expected to promote immune adaptability and to direct immune-related clinical outcomes. Interestingly, participants in Schalow’s trial demonstrated a significant (p<0.01) and transient 117.85 mg/ml increase of mean SIgA levels 30 minutes post-adjustment as compared to pre-adjustment (mean pre-adjustment [M] = 311.05, SD = 202.37), mean post-adjustment [M] = 428.90, SD = 329.70); endpoint measurement two weeks post-adjustment revealed restoration of SIgA levels to indistinguishable from baseline pre-adjustment [141]. These data demonstrate a transient increase in an indicator of systemic immunity, consistent with a post-adjustment “boost” in immune adaptability. Consistent with this observation, changes in most cytokines and immune modulators are transient by their nature and purpose [277]. Although randomized studies (particularly studies in patients with symptomatic illness) will be necessary to confirm and expand on this finding, the results of Schalow’s observational study provide translational research findings connecting the existing body of research on chiropractic care-mediated modulation of NEI system parameters to a quantitative impact on a clinically significant outcome measure, predictive of improved immune outcomes.

In sum, current evidence demonstrates that chiropractic care can impact a range of immune and neuroimmune biomarkers, potentially explaining the observations of immune function improvements reported by field practitioners in case studies. The possible biological mechanisms by which VS may impact known NEI modulation pathways are multiple and overlapping, including but not limited to evoking functional changes within the cholinergic anti-inflammatory pathway [278], promoting population shifts within leukocyte populations [279], activation of immune system effectors [239], and indirect effects on immune function as would be mediated by cortisol flux [230,280]. Future research should be directed at detailed characterization of the effects of VS and its reduction or resolution on the above parameters, in relation to overall clinical immune outcomes and in different populations, with the goal of enhancing the current understanding of the relationship between chiropractic care and immune health and adaptability.

Interferon

The body releases immune-modulatory cytokines termed IFNs upon recognition of viral infection in order to upregulate the immune response. IFNs also play a vital role in tumor suppression and upregulation of MHC Class 1 and 2, and signal transduction [281]. At least one IFN, interferon-gamma (INFγ), whose receptors are expressed on most cell types, has secondary neuroendocrine roles outside of the immune system including regulation of adipocyte insulin signaling, lipid storage, and differentiation and modulation of neuronal function, connectivity, and social behavior [282]. IFNs are therefore effector molecules within the NEI supersystem, with multiple roles in integrative physiology.

The present search identified two papers [87,95] detailing the quantitative change in interferon levels with chiropractic care. The first, a 71-participant RCT employing in vitro analysis of the activity of biomarkers from blood cells isolated from cohorts of asymptomatic, chronic, or acute LBP patients, described a trend toward reduction of IFNγ production from cells isolated from SMT-treated patients with either acute or chronic LBP, but not asymptomatic patients, with a more pronounced effect observed in chronic LBP patients [95]. A more recent preliminary study [87] assessed IFNγ levels in 19 patients subjected to adjustments of differing force magnitude. The investigators found that while sham-treated individuals showed little short-term change in IFNγ levels, individuals subjected to a 400N force tended to decrease IFNγ levels while those subjected to an 800N force exhibited an increase of IFNγ immediately and 20 minutes post-adjustment. The authors of both papers hypothesize that inflammatory changes from spinal adjustment may be responsible for the alteration of cytokine levels, including IFNs. While these promising studies are preliminary, and do not account for VS status per level specifically, both provide an indication that IFNγ levels may change as a result of chiropractic care directed at the spine. 

As with cytokines discussed in the section above, changes to interleukins with chiropractic care may have a simple biomechanical etiology: mechanical deformation to fascia and/or matrix may evoke the release of small molecule messengers [242-244]. Future studies should be conducted to confirm or refute the early observations discussed above and to more specifically elucidate the contribution of VS and the specific chiropractic adjustment to observed outcomes. Serum multiplex testing may be useful in this endeavor. Furthermore, future research should be directed towards determining whether observed changes in IFNγ and other IFN levels are of such a magnitude or duration as to elicit changes in immune, neural, or endocrine processes or outcomes in vivo.

Interleukin

Interleukins were originally identified as cytokines that carry messages between white blood cells. The primary function of interleukins is to modulate growth, differentiation, and activation during inflammatory and immune responses [283] though certain interleukins are pleiotropic, including interleukin 6 (IL-6) [284], a “master player” amongst cytokines which can stimulate the HPA axis during inflammatory stress, can modulate both synaptic transmission and neuroimmune communication [279], and has multiple endocrine effects [285]. Interleukins, like IFNs, are therefore effector molecules with diverse functions within the NEI supersystem. Biologically, the flux of interleukins can be affected directly by spinal interventions via simple biomechanical stress [286,287] or may be altered by the presence or change of the inflammatory state of the vertebral motion segment [288,289].

In our literature search, 10 references [84-87,90-92,95,137,138] were identified offering quantitative exploration of changes in interleukin levels with spinal adjustment, including five original papers and five reviews. One recent systematic review and meta-analysis found moderate-quality evidence that spinal manipulation is better than control in influencing interleukin concentration [86], while another noted a trend that spinal manipulation reduced the levels of IL-1b compared with sham after an intervention period of three weeks [84]. Several of the identified RCTs offer in vitro evidence of changes in interleukin release in cells (including IL-2, IL-6, and IL-1β) isolated from patients subjected to SMT, as compared to control [91,92,95]; these data, though thought-provoking, have not yet been connected to impact on overall clinical outcomes in vivo. Of the remaining papers, data within one trial of 21 participants indicate a decrease in serum levels of IL-6 in the treatment group after nine sessions of instrument-mediated adjustment to the lumbopelvic hip complex [90]. Most recently, Duarte et al. [87] conducted an assessment of immediate changes in a select panel of blood biomarkers (pro- and anti-inflammatory cytokines) in 19 young healthy adults subjected to posterior-to-anterior thoracic (T6-T9) SMT using two different force magnitudes. Changes were noted for levels of interleukins IL-5 and IL-6: elevation of systemic pro-inflammatory interleukin IL-5 was noted 20 minutes post-intervention at higher force (800N, comparable to 180 lb of force exerted) as compared to lower force (400N, comparable to 90 lb of force, considered "moderate"), while IL-6 increased immediately post-intervention in the 800N subjects as compared to 400N force. Of note, in the 800N group, both IL-5 and IL-6 levels trend towards increase, whilst with 400N force delivery levels of these interleukins trend towards decrease during the same time frame. As with previously discussed keyword searches, one notable weakness in the available body of literature exploring the effect of chiropractic care on interleukins is the lack of categorization of patient cohort VS status. Future studies should include the separation of study cohorts into patients bearing VS, per level, versus sham control patients not exhibiting VS.

Considered in sum, the above data provide supportive evidence that spinal adjustment can transiently and/or immediately modulate serum interleukin levels. Whether this yields meaningful impacts on or clinical outcomes of NEI processes or pathologies remains to be explored. With the increased commercial availability of multiplex testing methods, future studies are recommended to test for alterations in an array of interleukins measuring short and long-term changes post-intervention. Such considerations would be expected to be important for a group of cytokines whose actions are transient by their nature [283]. Similarly, future research should be directed towards exploring potential clinical impacts of interleukin flux caused by chiropractic care, such as acute and longitudinal modulation of the inflammasome and its effects on immunity and pathology [290,291].

Sleep

As a biological process, sleep is an essential element for overall health and function, and disordered sleep is considered a growing public health concern [292,293]. Disordered sleep is considered a foundational contributing factor to multiple functional abnormalities in the body, including dysregulation of growth hormone, cortisol, and the HPA “stress” axis [294,295]. Therefore sleep influences broader dysfunctions within the NEI supersystem [296,297]. Abnormal sleep patterns have metabolic, cardiovascular, immune, and inflammatory consequences [298-300] and are correlated with an increased risk of all-cause mortality [301,302]. Sleep disorders are related to autonomic dysfunction via a bidirectional relationship, and sympathetic activation may interfere with the brain’s transitions between sleep stages [303,304]. Any intervention that breaks the vicious cycle of this bidirectional relationship would be expected to enhance overall health and function. Sleep therefore represents both a powerful modulator of NEI function and a clinically relevant outcome of care, unto itself, as a biological process. Restoration of normal sleep patterns would therefore represent a salutogenic process [28,29] as such improvement would be expected to elicit a positive change in the health ease/disease continuum.

A collection of 29 published papers identified by the present literature search supports the contention that chiropractic care quantitatively restores or improves sleep [146-174], including 20 case reports, eight trials, and one review. Of this collection, 18 case studies were identified that documented improvement in total hours of sleep per night or number of sleep interruptions. The majority of case studies identified describe the benefits of correction or reduction of VS for improvement of sleep in infant and pediatric populations [146,147,149,154,158,161,162,167,169-171,174], while several case studies document similar improvements in adolescents and adults [151,153,155,156,159,173]. Eight trials (primarily observational, pilot, prospective, or survey-based comparative studies) outlining the quantitative benefit of spinal manipulation therapy for various aspects of sleep were identified in our search for the infant population [148,163-165], pregnant women [172], individuals with fibromyalgia (FM) [157,166], or in a normal asymptomatic adult population [150]. Limitations of these data include lack of RCTs in different populations to explore the effects of chiropractic care on subjects in different eras of life, and lack of specific VS characterization of subjects in many of these studies. The observational and clinical data discussed above comprise preliminary foundational evidence supporting the hypothesis that chiropractic care can improve the biological process of sleep.

Future studies should be aimed at elucidating what types of sleep challenges respond to chiropractic care, in which populations, and whether any specific VS patterns are causative in the etiology of sleep difficulties. A related aspect of sleep biology that may be particularly pertinent to future research by the chiropractic profession is the relationship between sleep and the glymphatic system, which clears out metabolic wastes from the brain during normal sleep processes. Glymphatic system function appears to be active primarily during sleep and follows a circadian rhythm [305]. Impaired glymphatic clearance, as has been hypothesized to result from VS at C1/atlas in particular [306], has been implicated in Alzheimer’s disease and other degenerative pathologies [307,308]. Restoration of normal sleep patterns via chiropractic care could reduce the incidence and prevalence of these and other neurodegenerative diseases, lessening the global social and economic burden of these pathologies, and therefore this area of study merits further exploration from a public health perspective.

Sleep and its benefits may be impacted by multiple factors or etiologies arising from the presence of VS, and this relatively new concept promises to be an active area for chiropractic research in the future. The biological mechanism by which chiropractic care impacts sleep biology may be autonomic in nature. Chiropractic care can produce effects on both the parasympathetic and the sympathetic nervous system [309]; indeed, many of the beneficial outcomes of chiropractic care published in literature, including a decrease in anxiety [113,156], improvement in HRV [104,119,121], and normalization of blood pressure [46], may reflect a common etiology of normalization of sympathetic outflow. Restoration of normal sympathetic and parasympathetic flux may promote ease of transition between sleep stages, breaking the aforementioned vicious cycle and eliciting an outcome of improved sleep, which would in turn be expected to evoke broader effects on the body. Future studies of potential impacts of chiropractic care on sleep parameters may include monitoring by polysomnography in sleep lab settings; alternatively, cohort longitudinal observational studies may be approached by participant use of "wearable" devices such as the Oura ring, which have high accuracy of four-stage classification (light, deep, rapid eye movement (REM), and wake) compared to gold-standard polysomnography [310].

Substance P

Although the substance P was originally considered primarily a neurotransmitter and effector of musculoskeletal disorders, it is also a key molecule in neurogenic inflammation response, regulating cell functions by autocrine, paracrine, endocrine, and neuroendocrine mechanisms [311]. Substance P is secreted and synthesized by nerve cells as well as many types of immune cells, is present in both CSF and blood, and has roles in a diverse array of physiological processes including control of inflammation [312], regulation of other cytokines particularly in the GI tract [313], governance of bone homeostasis [314], regulation of cardiac inflammation [315], modulation of inflammatory CNS disorders [316], and it influences anxiety behaviors via effects on the sympathetic nervous system via activation of the HPA axis and subsequent release of cortisol [317]. Substance P therefore represents another small molecule effector within the NEI supersystem that may impact a wide variety of physiological outcomes.

The impact of chiropractic care on substance P has been examined in four reports [86,175-177]. Two reviews [86,175] offer consideration of the three available early original research studies (including Brennan et al. [318] which is not specifically included within the present dataset since it was published in 1991) and discuss limited evidence that substance P increases after spinal manipulation. Of the available original research studies meeting our inclusion criteria, two describe positive immediate and short-term changes in substance P after spinal manipulation [176,177], and one paper was identified in our search that describes no change in substance P [92]. Further research will be necessary to clarify this apparent discrepancy; however, the results observed in the two former studies suggest that examination of changes in substance P levels in response to chiropractic care merits further consideration.

Importantly, the two studies describing changes in levels of substance P after chiropractic care measured not local, but global (serum) changes. Since systemic substance P is a known mediator of NEI processes, these early data suggest the possibility that substance P may be one of a growing array of neuroendocrine mediators altered by chiropractic care.

As with cytokines, interleukins, and IFNs discussed previously, changes to substance P with chiropractic care may have a simple biomechanical etiology [242-244] with both local and systemic effects possible. Future studies should be conducted to confirm or refute the early observations discussed within this section, and to more specifically elucidate the contribution of VS and the specific chiropractic adjustment to observed outcomes.

Tumor Necrosis Factor-Alpha (TNF-α)

The pleiotropic inflammatory cytokine TNF-α is released by macrophages, NK cells, and lymphocytes and is central in orchestrating the inflammatory immune response [319]. Implicated in pathologies such as rheumatoid arthritis, ankylosing spondylitis, and Crohn’s disease [320], TNF-α also plays a role in neuropathic pain and neuroinflammation [321] and, therefore, this small molecule messenger functions as another channel of cross-communication between the nervous system and the immune system.

Six studies meeting inclusion/exclusion criteria for our database search exploring the impact of chiropractic care on TNF-α [88,89,92,95,96,178], including one case report of two patients, four trials, and a review. Early studies approaching the question of whether chiropractic care impacts levels of TNF-α [89,92] have yielded conflicting results. Zhang and Yao [96] proposed a putative biphasic TNF-α response to the SMT concept to resolve apparent conflicts in these reported observations and interpretations. Methodological differences should also be noted between the three studies described in this review, such as reported increased TNF-α levels in an in vitro study of blood isolated from healthy individuals [322] as compared to the observed reduction of TNF-α in individuals in the remaining two studies of symptomatic and asymptomatic patients, as assessed by serum levels and in vitro methods respectively [89,92]. Since Zhang and Yao’s 2016 narrative review, two additional studies have been published. One non-RCT reported that acute or chronic LBP patients experience a statistically significant decrease in in vitro TNF-α production from blood cells isolated post-manipulation, as compared to asymptomatic controls [95]. Another recent cohort study also described the reduction in elevated urinary TNF-α levels after a course of care for LBP, as compared to the control cohort [88]. Due to the significant methodological differences in this group of studies, no conclusion can be drawn as to whether TNF-α levels may trend upwards or downwards with adjustment; however, taken together, these studies certainly suggest that TNF-α can change in response to chiropractic care. The potential contribution of VS correction specifically to the alteration of TNF-α levels has not yet been adequately explored, and will be an area for future study.

TNF-α is known to be elevated in response to tissue injury [323] and altered levels may be present with vertebral subluxation and/or other spinal abnormalities or pathologies [324]. A reduction in TNF-α after chiropractic intervention may reflect decreased mechanical stress on the neural and structural tissues of spinal motion segments [325,326]. Additionally, change in urinary levels of TNF-α suggest the possibility that resolution of spinal pain or mechanical stress may contribute to a more global change in TNF-α levels. Indeed, elevated serum TNF-α is a marker for systemic inflammation, and over-expression of TNF-α is found in many acute autoimmune disorders [327]. A decrease in TNF-α may favorably impact the inflammation complicating autoimmune pathology, and TNF-α is currently a drug target for that purpose [328]. Whether chiropractic care reproducibly lowers TNF-α in symptomatic patients, and whether this impacts other processes, is speculative and may be the subject of future studies. Furthermore, future assessment of potential effects of chiropractic care on TNF-α may be conducted simultaneously with testing of other inflammasome-active small molecule messengers such as interferons and interleukins, via serum multiplex testing to assess potentially coordinated changes amongst these NEI modulators.

Discussion: Chiropractic care and integrative physiology

Research in recent decades has demonstrated that no part of the nervous system, central or peripheral, somatic or autonomic, can be considered independent of the immune system or the endocrine system. These three systems are linked to one another and to other systems inextricably, both directly and indirectly, and only together do the individual systems of the body create the symphony of dynamic adaptation and physiological flux that defines optimal functional health and wellness.

The present work represents an endeavor towards considering chiropractic research through the lens of integrative biology. Herein we consider the intersection of multiple subject areas within the current body of chiropractic research in the context of function within the established NEI supersystem. It should be noted that this supersystem unto itself is a member of a larger supersystem, and its interactions with the microbiome, and that biological effects on the NEI supersystem should in turn be considered in light of the fully integrated systems of the human body and its overall function and outcomes.

The research works considered here comprise a web of evidence that supports the contention that chiropractic care delivered to correct or reduce vertebral subluxation may have a quantifiable impact on mediators and outcomes of the NEI supersystem. The ability to flux within normal physiological parameters is a requisite property of adaptive systems, and any influence that diminishes the bandwidth of this flux would be expected to diminish adaptive efficiency under allostatic load. While many individual findings considered herein do not directly connect changes in specific lab values or mediators to clinical outcomes, the impacts on the outcomes of blood pressure, heart rate variability, immunity, and sleep demonstrate each represent improvements in specific aspects of adaptive efficiency within the NEI supersystem. Considered together, these basic science, observational, and clinical findings support a biologically plausible model in which the deleterious impact of vertebral subluxation on adaptive capacity can be mitigated via the chiropractic adjustment. The evidence discussed herein also helps to build a case that a somatic intervention, through afferent input into the central nervous system, can facilitate global changes through the interconnected nature of the NEI supersystem.

Connectomics as a framework for understanding NEI outcomes of chiropractic care

Connectomics is a vital and burgeoning field within neuroscience that considers interrelations between the functional and anatomical networks of neurons that comprise the brain and peripheral nervous system. Mapping interrelated neural networks via the Human Connectome Project and mesoscale connectomics [329-331], and concurrently employing both advanced imaging techniques and transneuronal tracing, has given scientists and researchers new and unexpected insights into both normal nervous system function and disease pathology [332,333]. The field of connectomics continues to expand the a priori knowledge base of neural connectivity by exploring the relationship between brain structure and function at the global level, but with neuron resolution [331,334]. Importantly, the approaches established within this new field of study bring forth an opportunity to embark on the next step of human neuroscience: exploration of how different neural subnetworks co-mingle and may be functionally coupled to influence and coordinate physiological adaptive responses. 

The promise of application of connectomics to our understanding of human neuroscience as a whole and the impact of chiropractic care specifically is an expansive one. Early discoveries in connectomics included paradigm-shifting findings such as the discovery that the human cortical visual connectome anatomically consists of three visual pathways, rather than two as previously thought [335]. These first explorations of connectomics also brought forth a striking suggestion that the cerebral cortex, the basal ganglia, and the cerebellum, previously thought to perform largely separate tasks, form an integrated and segregated network coordinating multiple motor and non-motor functions [336]. Similarly, connectomics research has elucidated a previously unknown direct motor-limbic interface [337], and has identified connections by which the sensorimotor cortex may modulate the adrenal medulla to support the coordination of hormonal and muscle responses to stress [338-340]. Connectomics-centered research has also identified top-down connections from the somatosensory, primary motor, insular, and medial prefrontal cortex that modulate sympathetic and parasympathetic control of the stomach [341]. Taken together, these findings represent unexpected fruits of the exploration of the field of connectomics: evidence of previously underappreciated mechanisms by which somatosensory afferents as a genre may directly influence or modulate other body processes such as autonomic function, a topic pertinent to the present integrative review. 

It is of note that the daunting task of mapping the structural and functional connections of the nervous system is eclipsed by the next level of study: exploration of neuromodulation of the neural network as a whole [342,343]. The study of neuromodulation and its impact on neuroplasticity of the human connectome is relevant in the discussion of the NEI supersystem as it relates to physiological resilience and adaptability. The interrelated nature of physiological systems including the neuroendocrine and immune systems is indeed reflected here: the next frontier, functional connectomics, may present the opportunity to directly observe the effects of neuromodulation within the NEI via small molecules produced by any of the subsystems, ranging from neurotransmitters to interleukins or interferons and hormones. 

Functional connectomics and the central autonomic network (CAN): A role for somatosensory afferent influence in cardioautonomic adaptability

An early entrant to the field of connectomics, the CAN is a functional network of interacting brainstem and forebrain components including the insular cortex, amygdala, hypothalamus, periaqueductal gray matter, parabrachial complex, nucleus tractus solitarius (NTS), and ventrolateral medulla [344]. Additional contributing elements more recently identified via fMRI include the thalamus, medial prefrontal cortex (mPFC), precuneus, anterior cingulate cortex, visual cortex, primary motor cortex, and cerebellum [345]. Together, these components comprise an integral modulator of the body’s adaptive response to internal and external challenges, receiving sensory input from numerous sources and directing changes in visceromotor, neuroendocrine, and behavioral responses in order to maintain homeostasis. The CAN is a central player in the neurovisceral integration (NVI) model of adaptation, which postulates that neural structures mediate a functional connection between cognitive/emotional regulation and cardiac vagal tone as indexed by HRV and establishes a connection between stress and physical health [257]. Significant anatomical and functional overlap is evident between the CAN and the Vagus Afferent Network (VAN) [346], which supports the importance of the CAN for vagally-mediated cholinergic and sympathetic immune modulation [278]. In light of the Human Connectome Project and advances in neuroimaging techniques, the CAN is receiving renewed attention [345,347], and researchers are beginning to elucidate both top-down descending cortical/central command elements and bottom-up signals ascending to the brainstem that can influence and modulate autonomic responses [347-349].

Several structures within the CAN that may mediate and/or integrate somatosensory influence on autonomic output have been identified, including the NTS, CAN forebrain structures, and the cerebellum. First, animal studies of the NTS, central to the CAN as well as the VAN, have demonstrated that this structure receives axons from both general somatic afferents (GSA) including spinal dorsal horn neurons that transmit sensory input from Aδ and C fibre (type III and IV), and viscerosomatic afferents (GVA) including primary baroreceptor afferents [350]. Importantly, the NTS was positively identified as a site for integration of general somatic and visceral somatic information due to the observation that sensory input from skeletal muscle contributes to functional alterations in the arterial baroreflex during ambulation [223,350,351]. In similar animal studies, the NTS has been shown to receive afferent input from upper cervical afferents via the intermedius nucleus of the medulla [219,352] and these cervical somatosensory afferents have been shown to affect autonomic function [220]. In human studies, type III and IV skeletal muscle afferents inform the exercise pressor reflex, by which somatosensory information from skeletal muscle modulates ANS-mediated peripheral vasoconstriction [353], and skeletal afferents are also known to be functionally involved in cardiovascular and ventilatory modulation via the NTS in the exercising human [354]. Together, these observations suggest the central importance of the NTS for integration of general somatic and visceral somatic afferent inputs for the generation of autonomic output. 

Forebrain structures can also mediate integration of somatosensory information with ongoing autonomic control of cardiovascular function. The insula and amygdala, in particular, are involved in integration of passive somatosensory input with baroreceptor information [355]. Similarly, small-fiber muscle somatosensory pathways have also been identified by fMRI within the supramedullary CAN in human subjects [356,357]. Passive electrical stimuli to type I and type II (Aa, Ab) somatosensory afferents of the forearm were shown to be processed by the left posterior insula and to impact the ventral medial prefrontal cortex and subgenual anterior cingulate cortex, suggesting that integration of sensory information from proprioceptors, static touch receptors, and joint stretch receptors may be associated with ANS-mediated suppression of cardiovascular state by way of CAN forebrain modulation [356,357]. Consistent with this data, the experimental application of repeated passive somatosensory input to congestive heart failure patients improves arterial baroreceptor sensitivity [358]. Cerebral structures have also been recently shown by transneuronal tracing studies in animal studies to directly control parasympathetic output to the stomach via multisynaptic connections [341], and neuronal connections have also been elucidated between both the motor cortex and the somatosensory cortex and the kidney, suggesting descending control via central command [340,359]. Similarly, the adrenal medulla, a major sympathetic effector, may be controlled by a multisynaptic series of neurons that connect it with the motor cortex, a finding that provides a putative link between body movement and modulation of stress [340]. These observations and other recent advances [360] suggest the potential importance of integrated somatosensory information, such as might be generated by spinal movement or impacted by vertebral subluxation, for the motor and somatosensory cortex-mediated modulation of autonomic responses.

Lastly, cerebellar relay provides a third source of somatosensory representation and integration within the CAN. The aforementioned NTS receives cerebellar proximal somatosensory input from the neck and trunk via the Fastigial Nucleus (FN) [361]. Direct connections also mediate communication between the cerebellum and the hypothalamus have been described which are implicated in somato-visceral integration via affecting both autonomic function and immune function [336,362-364].

An important question to consider is whether autonomically-mediated alterations in organ function can be traced backward to somatosensory modulation of the effector arm of the neural adaptation loop, or motor cortex modulation, given the reciprocal nature of connections between the sensory and motor cortices [365]. Recently, RV-mediated transneuronal tracing has been employed in animal models to elucidate the multi-synaptic neural circuits linking higher brain function to autonomic output and organ function [366]. RV-mediated transneuronal tracing in animal models has clearly demonstrated descending influence of motor cortex on adrenal medulla [338]. Pertinent to the chiropractic field, this output to the adrenal medulla originates largely from trunk and axial areas, suggesting a link between cortical control of “core” muscles and regulation of sympathetic output [340]. Similarly, both motor and nonmotor cortical areas were shown to contain output neurons that indirectly influence kidney function [359]: Specific cortical areas implicated include the primary motor cortex, primary somatosensory cortex, insula, and mPFC. Interestingly, the authors noted that these connections also originate predominantly from trunk representations within these cortical areas, and therefore the authors of this work have proposed a hypothesis that a map of visceromotor representation may be embedded within the classic homuncular map of skeletomotor representation [359]. A third line of experiments has recently outlined cortical influence over both sympathetic and parasympathetic arms of control of stomach function. Cortical neurons influencing sympathetic output to the stomach were found to originate from the primary motor cortex and primary somatosensory cortex, regions linked to skeletomotor regulation and control, and similarly, cortical neurons that influence parasympathetic output to the stomach were found to originate in the mPFC and insula [341]. While multisynaptic connections linking cortical areas to modulation of autonomic function have not yet been confirmed in human studies, the animal study findings above clearly indicate the importance of both sensory and motor influence over autonomic modulation of organ function in mammalian systems and indicate the need for further exploration of this phenomenon and its potential implications for human health. 

Taken together, these diverse studies clearly indicate that multiple and overlapping spinal and muscle somatosensory afferent pathways, of all fiber types, are hard-wired into the CAN and vagus afferent network, via the NTS and other portals of entry, and can inform or influence autonomic control of bodily organs under different conditions. These studies demonstrate that the somatosensory division of the nervous system cannot be considered as separate from the autonomic division: somatic afferent information is represented and can be considered in generation of an autonomic response at multiple levels. Since the ANS directly regulates immune system function, by both sympathetic and parasympathetic pathways [367,368], multiple forms of somatosensory and viscerosensory information must be integrated in order to coordinate a broad-scale NEI response to a stress or challenge. The relative importance of somatosensory afferents to integrated and coordinated adaptive responses has not been explored at this level of detail at this time, and this concept comprises a potentially interesting field of exploration. 

Vagus nerve stimulation (VNS): Clinical evidence that somatosensory afferent input can evoke integrative endocrine and immune responses and influence clinical outcomes

VNS, the modulation of vagus nerve efferent activity by electrical somatosensory afferent activation, has been explored for a century for its physiological benefits and was approved as neuromodulation therapy in 1997 for the alleviation of seizures [369,370]. Traditional VNS has been demonstrated to evoke an array of outcomes within the NEI axis, including improvement of chronic pain syndromes [371], improvement in depression [372,373], prevention of epileptic seizure [374], alleviation of irritable bowel disease [375] and Crohn’s disease [375], reduction of inflammation in rheumatoid arthritis [376] and modulation of splenic function [368]. The observation of measurable, repeatable, diverse physiological outcomes resulting from stimulation delivered to the vagus nerve, including somatic afferent fibers, underlines the concept that modulation of somatosensory afferent information represents an underappreciated and important opportunity to influence salutogenic adaptive responses in the body.

Only recently has a non-invasive alternative to traditional VNS, transcutaneous VNS, been explored as a therapeutic neuromodulatory intervention [377]. While traditional (invasive) VNS requires surgical implantation of an electrical neuromodulator directly connecting with the vagus nerve, newer, non-invasive modes of VNS include cervical VNS (cVNS), and transcutaneous VNS (tVNS, including auricular transcutaneous VNS, taVNS). The growing field of taVNS, in particular, capitalizes on Arnold’s nerve (also known as the auricular branch of the vagus nerve) that serves the pinna and/or tragus of the ear, sending general sensory afferent input (GSA, via fiber-type Ab [378]) to the vagus afferent network via the NTS [379,380]. The overall neural pathways of taNVS are similar to those activated by either traditional or cVNS, as confirmed by fMRI [381,382]. Preliminary taVNS research appears to echo established outcome findings of VNS, but with an important distinction relevant to this paper’s considered topic: these outcomes are elicited with general somatosensory input alone rather than via visceral/interoceptive pathways. Outcomes observed for taVNS include modulation of depression [383,384], migraine [385,386], and GI function [387], modulation of sympathetic nervous system activity [388,389], and recently, modulation of cytokines and chemokines in RA patients [390]. That general somatosensory afferent input elicits quantitative, reproducible, diverse outcomes not limited to the somatosensory system provides support to the contention that somatosensory input modulation by vertebral subluxation and its correction by the chiropractic adjustment may have farther-reaching effects than simply neuromusculoskeletal.

Electrical neuromodulation of vagus nerve activity is currently being studied in over 200 clinical trials for the treatment of a number of NEI conditions related to vagus nerve activity. The goal of this form of bioelectric medicine is to restore normal, healthy patterns of downstream nerve impulses by modulating input to the Vagus nerve [391]. This basic premise of VNS, that input to the brain can modulate output from the brain, is consistent with Kent’s proposed biological mechanisms of action of vertebral subluxation theory [181], in which disturbance to normal afferent input to the brain via VS is hypothesized to elicit abnormal output from the brain (effector functions). The efficacy of VNS as observed by its clinical outcomes, then, may provide a biologically plausible explanation for the observed effects of correcting vertebral subluxations and thereby restoring normal afferent input to the brain, eliciting a change in effector functions dictated by the brain.

One important question with respect to taVNS somatosensory afferent input is the exact nature and functional role of the observed GSA-mediated activation of the primary somatosensory cortex (S1): does this input directly or indirectly influence the autonomic nervous system? Currently, it is unclear whether such activation of S1 is directly/causally related to ANS modulation, or whether S1 activation with taVNS is a less relevant by-product observed after activation of brainstem (NTS) and other cortical structures, though these possibilities are not mutually exclusive. Three observations suggest that GSA can also modulate ANS activity directly via S1. First, the pressor reflex or muscle-specific nerve activity (MSNA), by which skeletal somatosensory afferents influence sympathetic vasomotor outflow [353,392], demonstrates the importance of functional coupling of somatosensory information to modulation of sympathetic outflow. Interestingly, the pressor reflex was replicated using taVNS [393] rather than electrical stimulation of muscle afferents, suggesting overlap or confluence of these sensory inputs. Second, median nerve somatosensory evoked field(s) have been shown to influence response to VNS: in a cohort study, functional connectivity of sensorimotor and limbic areas activated by median nerve activity was shown to predict response to VNS [394]. Third, functional connectivity studies on fibromyalgia (FM) patients have demonstrated functional alterations in somatosensory connectivity and have shown that painful somatosensory stimulation decreases autonomic output (HF-HRV) [395]. Taken together, these findings underscore that somatosensory afferent input, via the NTS, the primary somatosensory cortex S1, or both, is a causal rather than simply temporally correlated factor in VNS-mediated autonomic modulation. 

Supporting the nascent concept that multiple modes of sensory input can modulate autonomic outcomes, it is of particular interest that afferent input from CAN and VAN converge to common neural signaling pathways. Both networks may be activated by Ab fiber somatosensory input via the NTS, and therefore observed outcomes could be attributed to activation within either the CAN or the VAN, providing a form of overlap and redundancy commonly found in integrated systems. The pathways activated by taVNS have been mapped by multiple methods and provide a clear example of where somatosensory stimulation can evoke NEI outcomes. Thus, tVNS as well as other non-invasive bioelectric stimulation methods can serve as a proof of concept demonstrating how a somatosensory modality can have neuromodulatory effects on the NEI axis and affect neuroimmune outcomes, and may help to explain the somatovisceral effects observed with manual approaches described in the present review. tVNS also provides research methodology to test if manual approaches have similar or adjacent effects in connectomics research. 

The chiropractic connection: Neurostructural function, somatosensory afferents, and coordinated adaptation

**Figure 2:**
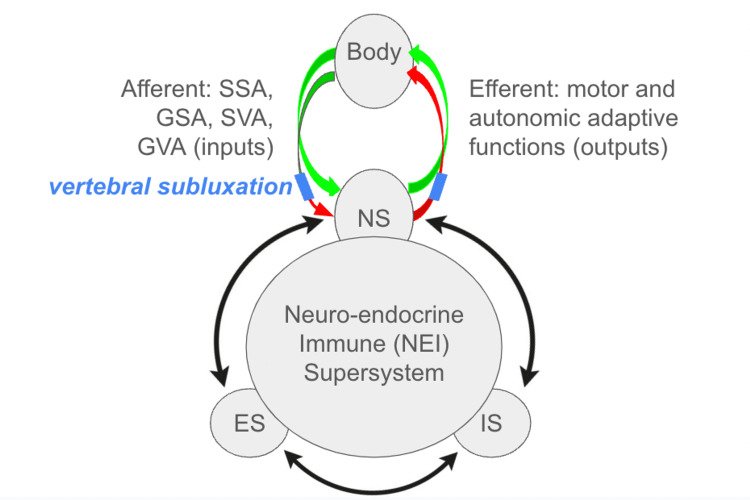
Theoretical model of the impact of vertebral subluxation on neural information flow and on the extended neuro-endocrine immune supersystem. Green arrows represent normal afferent and efferent neural information flow; red arrows indicate altered afferent or efferent flow via vertebral subluxation (blue box). The chiropractic adjustment delivered to eliminate or reduce vertebral subluxation would restore normal afferent/integrative/efferent flow (green arrows). NS, nervous system; ES, endocrine system; IS, immune system; SSA, somatosensory afferent; GSA, general somatic afferent; SVA, special visceral afferent; GVA, general visceral afferent

Through the lens of dynamical and integrative systems biology [396] this paper presents a theory that chiropractic care delivered to eliminate or reduce vertebral subluxation (represented by blue box, Figure 2), which alters quantity, quality, or nature of afferent or efferent input (red arrows), would restore the integrity of information flow between the body and the central nervous system (upper green arrows). By reconstituting normal information flow and integration, the specific chiropractic adjustment would promote restoration of appropriate adaptive commands sent from the nervous system to the body/periphery. Efferent outputs include both motor and autonomic adaptations, which in turn could impact immune or endocrine functions, given the interconnected nature of these systems.

The role of spinal manipulation or chiropractic adjustment evoking somatosensory afferents that affect afferent function within the central nervous system has been explored and demonstrated in both animal and human models [397-399] and an established body of research has elegantly described how chiropractic adjustments or SMT can generate quantifiable changes in neurophysiological activity at both the spinal and supraspinal levels [191,192,400-406]. These changes include improvements in movement time, muscle contraction strength, somatosensory integration, joint position sense, cortical drive, and spinal motor neuron excitability. Taken together, these findings demonstrate that either chiropractic adjustment or SMT to the spine evokes somatosensory afferent input that can alter neural output. It follows, then, that both vertebral subluxation and its physical correction via chiropractic care may alter quality or quantity of afferent input, with downstream physiological effects. Whether autonomic or visceral effects of any specific, per-level vertebral subluxation are subclinically or clinically relevant, or whether correction of that putative neuro-spinal abnormality will have predictable clinically meaningful outcomes, will be areas for future study. 

Current chiropractic research methods concerns and future directions

As with any nascent field of study, further research will be necessary to comprehensively trace causative and directive changes eliciting NEI clinical outcomes via chiropractic care. To facilitate this process, such research should incorporate updates to several methodological flaws commonly observed in currently available chiropractic research. The following research methodology concerns should be considered: (1) attention to data interpretability issues that may arise due to use of asymptomatic subject cohorts; (2) failure to consider vertebral subluxation as an experimental variable; (3) awareness of the consequences of inadequate sham controls, (4) simplification of study or trial design, to render studies more comparable to one another to allow for reliable meta-analysis; (5) incorporation of both pre-adjustment and post-adjustment measurements, of both vertebral subluxation indicators and clinical outcomes, to strengthen conclusions of temporal correlation or causation of correction or reduction of vertebral subluxation with the emergence of clinical outcomes; (6) study design that accounts for the possibility of either positive or negative change as compared to baseline value, rather than simply "improvement;" (7) clear delineation of whether participants and/or cohorts are taking medication(s) that may complicate interpretation of outcomes data; and (8) consistent use of specific and appropriate terminology in research reports in the future, so as to correct the historical conflation of the distinct terms "chiropractic adjustment" and "spinal manipulation." Since the level of detail of research method planning determines the quality of outcomes data, corrections to the following issues should be incorporated into future studies so that chiropractic research may yield the most interpretable and meaningful information possible.

First, the use of asymptomatic patients (“healthy volunteers'') as a study cohort brings forth a question as to whether a measured experimental parameter would be anticipated to shift away from a normal (“healthy”) baseline value, to yield quantitative outcomes or measurable results, with intervention. Complexity within a biological system is considered a ubiquitous phenomenon that allows living systems to adapt to external perturbations whilst preserving homeostasis [407]. While normalization of an abnormal parameter would certainly be hypothesized or anticipated in a cohort of symptomatic/abnormal study cohort, the entropy of a self-governing system within a normal cohort would be expected to utilize feedback loops to maintain a balanced overall constellation of biological setpoints with no net change (dynamic equilibrium). Induction of change from a normal setpoint towards a perturbed value may flow against the synergy of the overall system and may evoke an auto-corrective response towards restoration of the system’s normal setpoint, an outcome that would be observed as “no [net] change.” In the context of the present collection of research works, several papers describe use of asymptomatic, normal individuals as study participants. Examples include the use of normotensive individuals to study whether chiropractic adjustments alter blood pressure, use of healthy volunteers for HRV studies, and use of healthy, uninfected patients for cytokine research. Results from the aforementioned studies may therefore be skewed towards outcomes of no net observable change, which would lead to inappropriate confirmation of the null hypothesis. Perhaps research designed to explore whether chiropractic care affects flux of any physiological parameter may yield more meaningful results if the chosen parameter is abnormal at baseline value in a study cohort, rather than at a normal setpoint. Further, biological setpoints may differ significantly amongst "normal" participants, since a setpoint is typically a range plus or minus standard deviation, rather than a single value. Observational cohort or case studies in which individual patients, or a cohort of patients with similar baseline characteristics, may be compared to their own baseline value after intervention might be useful here to account for natural setpoint variability. Also worth considering is the possibility that within a given study, participants with the most extreme "abnormal" values would be expected to have more significant outcomes, simply from a mathematical standpoint. Therefore, inclusion of a cohort of participants with strongly abnormal baseline values for an experimental parameter, rather than "healthy volunteers," may be a more preferable criteria to yield significant or meaningful data.

Second, failure to assess for vertebral subluxation as a clinical rationale for providing a per-level intervention of spinal adjustment or manipulation constitutes an error that may lead to inappropriate use of an intervention or inappropriate location of an intervention. As discussed previously, to design studies appraising changes in neuroendocrine markers via chiropractic care presupposes the existence of a spinal condition (vertebral subluxation) that affects function within the NEI supersystem. If vertebral subluxation is the causative factor responsible for maladaptive neural changes that impact NEI supersystem flux, an adjustive thrust targeted at a vertebral segment without vertebral subluxation (as per delivery of spinal manipulation) may fail to correct a cause that is not present, leading to lack of measurable outcomes, or potentially worsening of outcomes. In other words, from a research methods standpoint, the functional condition of the vertebral level is more important than the spinal level of the delivered intervention per se for eliciting outcomes with the intervention. As such, the presence of vertebral subluxation in a study cohort presents a variable to characterize per level and per subject, lest it present a de facto confounding variable in both control and intervention arms. Vertebral subluxation at a segment is thought to create a physiological microenvironment with different tissue characteristics (including local inflammation and changes in local muscle tone that elicit alterations in central motor control [202]) as compared to a normal vertebral segment. Assumption of per-level equivalency without adequate objective characterization may unintentionally result in random presence or absence of vertebral subluxation(s) in either or both control and/or intervention arm, a classic confounding error in research methods. For these reasons, research that endeavors to explore the influence of a spinal abnormality (vertebral subluxation) whose adjustment may impact neuro-endocrine immune processes and/or outcomes should be carefully planned so as to appropriately control for presence or absence of VS in study cohorts, to ensure that outcomes are clear and not confounded. Incorporating the diligence of characterization of vertebral subluxation per level [182] in prospective participants allows for allocation of normative (displaying no vertebral subluxation at the vertebral level to be addressed) participants into a control cohort, to serve as a comparator for the effects of the adjustment, or no adjustment, on participants confirmed to bear vertebral subluxation per level. Vertebral subluxation has a considerable prevalence in the human population, as measured by MRI [183] and epidemiological [408] studies, and may be present at any or multiple spinal levels, underscoring the necessity of accounting for its influence in research methods design. Failure to account for and control for vertebral subluxation as a clinical variable therefore comprises a research methods flaw, correction of which will be necessary to generate clear and non-confounded data. 

Third, the availability of validated sham controls has long presented a research methods problem within chiropractic research. The primary challenges of sham selection in chiropractic research include using a maneuver that is indistinguishable from the intervention, and choosing a maneuver that does not elicit somatosensory effects that may overlap with those of an adjustment/manipulation. Unfortunately, an “adjusted” versus “non-adjusted” intervention is often easily distinguished by the patient in the case of traditional manual adjusting, impacting the viability of blinding/placebo control. Further, use of drop table mechanism without contact as a sham control for Toggle adjusting, or thrust without cavitation (or thrust on another segment) for traditional manual adjusting may elicit a response unto itself, and whether the sham intervention elicits a response from the body as compared to no intervention is often uncharacterized. 

Certain chiropractic methodologies for spinal adjustment may be better suited for research purposes. For example, the use of instrument and light force techniques allow for the use of sham control that may be more indistinguishable to a patient than a high-velocity, low-amplitude thrust. These techniques can further be studied by studying the impact of these techniques when applied on regions with and without vertebral subluxation in order to compare the neurophysiologic effects of the thrust as compared to the effects of correcting the spinal lesion. The employment of chiropractic adjustive approaches such as National Upper Cervical Chiropractic Association (NUCCA) or Atlas Orthogonal (AO) techniques, both of which utilize a light touch with vectored force that cannot be easily distinguished by trial participants from sham light touch without vectored force, offers a reasonable approach to satisfy the first condition. A second “sham” control, with no intervention whatsoever, may also be employed in future studies to discern whether the intended sham control elicits outcomes indistinguishable from light touch/no force sham. 

Fourth, clear interpretation of results yielded from any experimental system benefits from reduction of variables wherever possible. Vertebral subluxation can be present within the spinal column singly, or in any combination or permutation of multiple subluxations [183] and it is most often observed by clinicians as a pattern or constellation of subluxations, rather than affecting only one segment. The specific constellation of vertebral subluxations would be expected to differ from person to person, due to unique life traumas and microtraumas, experiences, and habits. Given the possible permutations of vertebral subluxations within the spinal column, identifying a cohort of individuals who bear the exact same vertebral subluxation pattern for clinical trial purposes would be anticipated to be challenging. Selecting one segment for experimental/adjustive intervention, rather than adjusting multiple segments of the spine during one chiropractic session, may yield more clearly interpretable results. Adding to this problem of variability, methods to analyze/detect and adjust vertebral subluxation are many and diverse within the chiropractic profession. The permutations and variability of vertebral subluxation analysis methods and adjustive interventions make comparability between results from different clinicians challenging. With these variability issues in mind, well-designed n=1 studies [409] (case studies) and case series reports provide a unique resource for reporting chiropractic outcomes. In n=1 studies, the study subject’s baseline before care for any measured parameter serves as an internal control/comparator, allowing the investigator to infer cause and effect from correction of vertebral subluxation, particularly if no other variables in the patient’s lifestyle (such as addition/subtraction of supplements, exercises) change between baseline and intervention. Additionally, if a patient achieves a measurable change with a series of adjustments but stops care and subsequently relapses to baseline condition, re-introduction of care with reproduction of the same outcomes as previous may speak to reproducibility of effects of the adjustment. Similarly, observational case series studies can follow objective changes in cohorts of participants bearing similar subluxation presentations over time, and these longitudinal observational studies can yield different insights than short-term studies. One must keep in mind the limitations of case reports, case series, and observational trials as lacking overall generalizability due to the potential presence of confounders, and one also must keep in mind that the quality of data obtained will always depend on what parameters were measured at baseline, since a baseline value cannot be obtained after care has been delivered. 

Fifth, many available studies fail to report reduction or resolution of the indicators of vertebral subluxation with chiropractic adjustment, choosing to report clinical outcomes only. The omission of a direct comparison of pre- versus post-adjustment indicators weakens conclusions that contend that clinical outcomes are a result of either spinal manipulation or correction or reduction of vertebral subluxation [410]. Notably, some investigators have incorporated pre- versus post-adjustment measurements of their vertebral subluxation indicators [46,48,141,156], which strengthens the conclusions of these particular studies. Disciplined reporting of both pre-adjustment and post-adjustment vertebral subluxation indicators is requisite for establishing temporal correlation of correction or reduction of vertebral subluxation with clinical outcomes, or establishing a causative link thereof.

Sixth, a study design that accounts for either positive or negative change of parameter as compared to baseline may be appropriate for assessment of clinical variables whose change may be positive, negative, or biphasic. Blood pressure, cortisol, and tumor necrosis factor have been discussed previously in this work as examples of parameters that may not necessarily change in mathematically positive direction; measurement of HRV may also benefit from a study design that teases out both positive and negative changes, both of which can be clinically significant [250]. 

Seventh, medication use constitutes an experimental study variable that can affect outcomes. Future studies should be designed to minimize the possibility that medication use might influence outcomes as a confounding variable, and published reports should clearly and transparently state whether medication use in the study cohort was specified as exclusion criteria.

Lastly, both theoretical and practical distinctions between the non-synonymous terms "chiropractic care" and "spinal manipulation therapy" have been outlined herein, and these distinctions have important implications to study design and data interpretation. Future research reports should take care to specify which mode of care was employed per study, in the interests of clarity, transparency, and integrity.

Toward realization of an EIP model of chiropractic care

The EBP paradigm was created as a means to normalize patient care in a given discipline according to findings of high-quality research. While its strengths include adoption of a universal standard of care via research-based guidelines per discipline, a limitation of EBP is whether the conclusions of available RCTs apply to the unique patient as presenting to the clinician. Often, lacking a perfect match between RCT inclusion/exclusion conditions and patient presentation, a clinician will apply an intervention “off-label,” which in effect is contradictory to the intended practice of evidence-based care. The quality and character of RCTs dictate their applicability and generalizability, and taken together these elements comprise the limiting factors for whether EBP can truly be followed. 

EIP was proposed as a modification of the concept of EBP, encouraging more reliance for clinical decision-making on all available evidence - not limited to RCTs but instead inclusive of observational studies such as case series, cohort studies, and case studies [411,412], and inclusive of both clinician experience and patient preference. Of note, the unique value of case studies is that the subject of a case study effectively serves as their own negative control pre- versus post-intervention, allowing for case studies to describe unique patient presentations such as a particular constellation of vertebral subluxations. Case-matched, well-done case studies can therefore be an opportunity for clinicians looking to glean potentially important clinical insights whilst in their analysis process [413]. The EIP model recognizes clinicians as critical thinkers and decision-makers. It allows clinicians to make appropriate care decisions based on multiple factors involved with patient care including but not limited to all available evidence, patient preferences, clinical circumstances/environment, clinician knowledge/experience, and clinician level of training which may not necessarily match the criteria outlined in a clinical trial [44]. Given the challenges of the widespread prevalence of neuro-spinal functional abnormality/vertebral subluxation considered in the context of the number of permutations/combinations of multi-level VS that may be possible within a population [183,408], strict EBP based on RCTs may effectively be of less utility and applicability as compared to the adoption of an EIP model, an approach that considers a wider swath of evidence inclusive of all available levels of evidence. Consistent with this possibility, it is important to bear in mind that the clinically observed benefits and outcomes of chiropractic care are person-specific, owing to factors affecting the unique individual, underscoring the premise that EIP represents a more appropriate construct for clinical decision-making in chiropractic care.

That chiropractic care impacts nervous system function and adaptability has been well-established; however, in recent years, certain entities within the chiropractic profession have endeavored to limit the scope of chiropractic to neuromusculoskeletal, musculoskeletal or pain-based only. This paper establishes the short-sightedness of such an impetus, and outlines an evidence-centered opportunity for exploration of the impact of chiropractic care on the connected systems of the human body. The integrative review presented herein describes a broad net of evidence that vertebral subluxation, and chiropractic adjustment targeted at the vertebral subluxation specifically, can modulate a variety of neuro-endocrine immune functional changes and outcomes. Unequivocal evidence “proving” conclusively that chiropractic care produces predictable, reproducible outcomes in all patient populations has not yet been fully elucidated, however the available evidence supports a role for chiropractic care for facilitating restoration of normal neuro-endocrine immune functions and outcomes. This includes, but is not limited to, normalization of blood pressure, augmentation of cardioautonomic adaptability as measured by HRV, modulation of immune response as measured by cytokine and antibody secretion, and improvement of the biologically requisite process of sleep which is in turn foundational for repair, regeneration, and maintenance of the human body. 

In an integrative physiology model, chiropractic care as discussed in this work may have wide-ranging effects on the coordination and adaptability of body systems beyond the musculoskeletal system with potential for short- and long-term salutogenic outcomes. The research question going forward is not whether chiropractic affects the NEI system, but rather to what extent, and under what conditions, with what impacts, on which clinical outcomes. Therefore in an EIP model, clinicians should be not only free but also encouraged to summarize any available research findings pertinent to any given clinical case, describe the findings of the studies (including strengths and limitations as well as applicability) in the context of their own clinical experience, and offer patients care options to consider. 

Furthermore, embracing an EIP model of clinical practice will protect and encourage the opportunity for observations to be made and may drive clinical research by field practitioners to continue advancing research and understanding of the potential benefits of chiropractic care delivered to address vertebral subluxation. Embracing EIP may transform the question of “How can chiropractic care benefit overall health?” to an updated question of, “How does vertebral subluxation interfere with normal human physiology, and how can chiropractic care be utilized to promote salutogenesis for the maintenance of healthy adaptability?” As such, this review contributes to the EIP model of chiropractic care, driving advances in patient-centered care. Future studies employing this upgraded clinical framework/paradigm will be requisite to investigate farther-reaching mechanisms toward our understanding of how chiropractic care for the location and correction of vertebral subluxation, can promote human physiological adaptability.

## Conclusions

Integrative physiology and connectomics have established the interconnected and interdependent nature of the body’s systems. While it has long been clear that chiropractic care impacts information flow and integration within the nervous system, current research suggests that consideration of broader physiologic effects of chiropractic adjustment on the NEI system is appropriate. The present analysis of literature outlines research findings that suggest chiropractic care may modulate NEI processes, including regulation of blood pressure, HRV, and sleep, as well as preclinical evidence of modulation of immune intermediates including cytokines and antibodies. Considered together via an inductivist approach, this collection of research suggests the possibility of chiropractic care for salutogenic facilitation of the body’s intrinsic integrated physiological processes by promoting aspects of neural adaptability and resilience, resulting in a positive shift along the body's health ease/disease continuum. Biologically plausible mechanisms by which VS and chiropractic care may modulate multiple physiological outcomes are discussed, drawing from established findings in the field of autonomic neuroscience, thereby strengthening our hypothesis. A noted limitation of this integrative review is that each subsection of articles discussed herein should in the future be adequately assessed via systematic and/or meta-analysis, to examine the depth of evidence within each category. Additional studies on each of the preliminary findings discussed herein will be necessary for such analyses, and future chiropractic research efforts should be planned carefully so as to avoid several research method errors that have complicated the interpretation of prior studies or their appropriateness for systematic or meta-analysis. The present work is offered as a framework for continuing reductionist studies that will be necessary to definitively establish a relationship between chiropractic care and modulation of integrative physiology via the NEI supersystem, and to elucidate precise neural mechanisms by which chiropractic care may elicit clinically relevant effects on integrative bodily functions. The present review also contributes to the practice of evidence-informed chiropractic care, driving advances in patient-centered care by establishing a compendium and analysis of studies for consideration by the chiropractic field practitioners as they navigate patient care.
